# Acupuncture for musculoskeletal pain: A meta-analysis and meta-regression of sham-controlled randomized clinical trials

**DOI:** 10.1038/srep30675

**Published:** 2016-07-29

**Authors:** Qi-ling Yuan, Peng Wang, Liang Liu, Fu Sun, Yong-song Cai, Wen-tao Wu, Mao-lin Ye, Jiang-tao Ma, Bang-bang Xu, Yin-gang Zhang

**Affiliations:** 1 Department of Orthopaedics of the First Affiliated Hospital, Medical School, Xi’an Jiaotong University, Xi’an 710061, Shaanxi, China; 2 Xi’an 521 Hospital, Xi’an 710065, Shaanxi, China; 3 Department of Orthopaedics of the First Affiliated Hospital of Xi’an Medical College, Xi’an 710077, Shaanxi, China; 4 Henan Province Hospital of TCM, Henan University of TCM, Zhengzhou 450008, Henan, China

## Abstract

The aims of this systematic review were to study the analgesic effect of real acupuncture and to explore whether sham acupuncture (SA) type is related to the estimated effect of real acupuncture for musculoskeletal pain. Five databases were searched. The outcome was pain or disability immediately (≤1 week) following an intervention. Standardized mean differences (SMDs) with 95% confidence intervals were calculated. Meta-regression was used to explore possible sources of heterogeneity. Sixty-three studies (6382 individuals) were included. Eight condition types were included. The pooled effect size was moderate for pain relief (59 trials, 4980 individuals, SMD −0.61, 95% CI −0.76 to −0.47; P < 0.001) and large for disability improvement (31 trials, 4876 individuals, −0.77, −1.05 to −0.49; P < 0.001). In a univariate meta-regression model, sham needle location and/or depth could explain most or all heterogeneities for some conditions (e.g., shoulder pain, low back pain, osteoarthritis, myofascial pain, and fibromyalgia); however, the interactions between subgroups via these covariates were not significant (P < 0.05). Our review provided low-quality evidence that real acupuncture has a moderate effect (approximate 12-point reduction on the 100-mm visual analogue scale) on musculoskeletal pain. SA type did not appear to be related to the estimated effect of real acupuncture.

Musculoskeletal disorders and the related pain are major causes of disability in both developed and developing countries^1^. Neck pain (NP), low back pain (LBP), osteoarthritis (OA), rheumatoid arthritis (RA), lateral epicondylitis, fibromyalgia (FM), and myofascial pain (MP) are common in our society^2^,^3^,^4^. Although mortality from these conditions is generally low, they have a major effect on disability, medical costs and patient quality of life, largely due to the associated musculoskeletal pain^5^. As the population continues to increase in age, the influence of musculoskeletal disorders on society will also increase. Currently, there is limited understanding of the mechanisms that cause musculoskeletal pain, and few therapies are available to treat musculoskeletal pain.

Acupuncture is commonly used for pain relief. The treatment is based on the theory that illness results from imbalances in energy flow, or qi, and fine needles are inserted at specific points on the body to correct these imbalances and restore harmony^6^. The incidences of side effects and adverse events with acupuncture are lower than that with opioid analgesics and anti-inflammatory medications^7^. Acupuncture has been claimed to be effective for a wide range of conditions, such as pain, musculoskeletal disorders and several neurologic diseases^8^. Gate control theory and the release of endogenous opioids have been suggested as explanations for the apparent analgesic effect of acupuncture^9^,^10^,^11^. Acupuncture has both physiologic and psychological effects^12^,^13^ that are described as either specific or non-specific. The specific effects refer to the analgesic effects produced by needling a specific site at a proper depth for an appropriate duration and number of treatment sessions. The psychological non-specific effects are associated with patient perceptions, beliefs, experiences, and expectations of patients. Therefore, sham acupuncture (SA) is needed to assess the specific effects of acupuncture. “Sham” or “placebo” is used to describe any control procedure that is used to blind treatment allocation in clinical trials of acupuncture^14^. Several sham procedures are now available, such as the use of penetrating acupuncture on non-acupoints, superficial penetration of the skin on acupoints and nonpenetration on acupoints with sham needle devices^14^.

Several reviews^15^,^16^,^17^ have evaluated the effects of acupuncture for musculoskeletal pain. However, all of them focused on only one disorder and almost all of them lacked analysis of the impact of SA type on the assessment of real acupuncture for musculoskeletal pain. Thus, we sought to analyze all previous studies of acupuncture for musculoskeletal pain that included a SA control group. Our objectives were to study the analgesic effect of real acupuncture and to explore whether SA type is related to the estimated effect of real acupuncture.

## Methods

This systematic review was registered with number CRD42014010760 (http://www.crd.york.ac.uk/PROSPERO).

### Criteria used to consider studies for this review

#### Types of studies

Only randomized clinical trials met our inclusion criteria. Both parallel and crossover studies were included. We included full articles with sufficient data for extraction, including the number of patients, the means and standard deviations for continuous outcomes in each group, and/or the number of patients in each group for dichotomous outcomes. There were no language restrictions.

Trials were excluded based on the following criteria: animal experiments, non-randomized or quasi-randomized (patients were allocated by registration number or date of birth) clinical trials, case report/series, news reports, letters, conference abstracts, or qualitative studies.

#### Types of participants

Patients suffering from pain associated with musculoskeletal disorders, defined broadly as pain that affects the muscles, ligaments and tendons, and bones, were included. The following conditions related to musculoskeletal disorders were included: OA, NP, LBP, cervical spondylosis, whiplash, shoulder pain (SP), lateral epicondylalgia, FM, ankylosing spondylitis, RA, gouty arthritis, and MP.

Patients with postoperative pain were excluded. Pregnant women with pelvic pain were also excluded.

#### Types of intervention

We pragmatically defined real (true, verus, genuine) acupuncture as an intervention in which needles were inserted into the skin at selected real acupuncture points at definite therapeutic depths. Trials with intervention groups that were treated with transcutaneous electrical nerve stimulation (TENS) or lasers were excluded.

#### Types of placebo

We defined SA as the use of “sham” or “placebo” needles. Sham groups exposed to sham TENS or lasers were excluded. We included trials that compared either acupuncture alone with SA alone or acupuncture plus one or more therapies with SA plus the same therapies.

#### Types of outcome measures

We only included studies that measured “follow-up pain or disability” immediately after the end of an intervention period (within 1 week) because studies with a shorter follow-up period would allow the detection of significant changes in pain. Our primary outcome was pain intensity (e.g., visual analogue scale, VAS; numerical rating scale, NRS; McGill Pain Questionnaire, MPQ). Our secondary outcome was disability (e.g., Oswestry Disability Index, ODI; Western Ontario and McMaster Universities Osteoarthritis (WOMAC) Index; Northwick Neck Pain Questionnaire, NPQ; Roland Morris Disability Questionnaire, RMQ). For each measurement, the closer the score was to 0, the more favorable the result.

### Search methods for study identification

We conducted our systematic review in accordance with PRISMA (Preferred Reporting Items for Systematic Reviews and Meta-Analyses) guideline^18^. We searched the following databases: MEDLINE, EMBASE, the Cochrane Library, the Traditional Chinese Medical Literature Analysis and Retrieval System (TCMLARS), the China National Knowledge Infrastructure (CNKI) and the Wan Fang database. The search was conducted from the inception of each database. No language or date restriction was applied. The reference lists of the included trials and previous systematic reviews were systematically searched for citations of potentially eligible trials. The authors of the articles were contacted if there were any questions about the trials.

Our search strategies were iteratively developed using ‘acupuncture’, synonyms of ‘sham’, ‘randomized clinical trial’, and ‘musculoskeletal disorders’ (see supplementary file).

### Data extraction, selection and coding

Identified studies were selected on the basis of titles and abstracts by two independent reviewers (QLY and MLY). Once a decision was made, full articles were checked. The kappa value statistic was used to measure agreement between the two reviewers. If there was any disagreement, either a consensus was reached or a third party (YGZ) became involved.

Two reviewers (QLY and LL) independently extracted the data from the studies using pilot-tested standardized data charts, and disagreement was resolved by negotiation or a third party (PW). Missing information was collected by contacting the corresponding authors of the studies.

The duration of pain was defined as follows: (1) chronic (≥3 months), (2) sub-acute (~1–3 months), and (3) acute (<1 month).

Primary outcomes included pain intensity (e.g., VAS and NRS) and disability (e.g., ODI).

We extracted and analyzed only comparisons that were based on outcomes measured immediately after an intervention (≦1 week); measurements taken more than 1 week after the end of an intervention period were not included in the analysis. We preferred post-treatment data (at the immediate term, ≦1 week) because follow-up data (>1 week) may be more prone to bias due to patients leaving a trial, the diminution of the effect and the few studies reporting a longer follow-up period.

The study details (author and publication year), treatments, conditions (e.g., OA, NP, and LBP), populations (demographic details), and outcome characteristics (including follow-up times) were summarized in tables.

The study (author and publication year), treatment, conditions (e.g., osteoarthritis, neck pain, low back pain), population (demographic details), and outcome characteristics (including follow-up times) were summarized in tables.

Specifically, the basic characteristics of acupuncture and SA were extracted according to Standards for Reporting Interventions in Clinical Trials of Acupuncture (STRICTA)^19^; these included theory of acupuncture, needle depth, needle location, name and number of acupoints selected, De Qi, and number and duration of treatment sessions.

For randomized crossover trials, only data from the first period were included because of the carry-out effect.

### Risk of bias (quality) assessment

Two reviewers (WTW and YSC) independently assessed the risk of bias in each study, and discrepancies were resolved by discussion or consensus with a third party (FS or BBX). The quality of each individual trial was evaluated according to the criteria of the Cochrane Back Review Group^20^. There were 12 items in total, and each item received 1 point for “yes” or 0 points for “unclear” or “no” (Supplementary Table S1). If the total score of a trial was equal to or larger than 6 points, the quality was considered high; a lower score would indicate low quality. The levels of agreement for each item and for the overall items were evaluated using the kappa value statistic.

### Strategy for data synthesis

The results were grouped according to condition (e.g., NP, LBP, and OA), pain persistence (e.g., acute, sub-acute, or chronic), SA type (e.g., needle depth or needle location), and trial location (based on continent).

The data were grouped into continuous and dichotomous variables and were pooled using a random effects model (DerSimonian-Laird method for standardized mean differences (SMDs), Mantel-Haenszel method for odds ratios (ORs)) to give a more conservative estimate of the effect of real acupuncture therapy on musculoskeletal disorders while allowing for any heterogeneity between studies. We preferred final values but used changes from baseline values only if these were the only available data. We preferred continuous data but used dichotomous data if the former were not available. We analyzed ordinal data as continuous data. If the means or standard deviations (SDs) were not reported and not available after contacting the authors, we used the data that were available, such as the median and its interquartile (IQR) or P values and confidence intervals, to calculate these values according to the methods recommended by the Cochrane Handbook, Version 5.1.0^21^. If mean values were reported without SDs, the SDs of baseline data were used. Engauge Digitizer 3.0 (by Mark Mitchell) software was used to extract data from figures for studies in which exact data were not shown in the text or listed in tables. Data acquired with these methods were verified, and only those data with the same direction of effect as the original article were included.

If the trials presented in a single paper included two or more real acupuncture arms or SA arms, the real acupuncture arms or SA arms were combined to avoid a unit-of-analysis error.

Heterogeneity between studies was evaluated using the I^2^ statistic with a cutoff point of ≥50%, and a P value <0.10 on the χ^2^ test was defined as a significant degree of heterogeneity.

Random effects univariate and multivariate meta-regressions were used to explore the source of heterogeneity if possible; this was accomplished by fitting covariables to participant details (i.e., age, sex, continent, baseline pain, acupuncture-naïve status, condition, and sample size); number of treatment sessions; treatment duration; sham needle location (i.e., same acupoints as real acupuncture, lateral to real acupoints, and acupoints of different or irrelevant conditions); sham needle depth (i.e., non-penetrating, penetrating superficially, or penetrating normally); trial quality (i.e., allocation concealment, blinding, use of intention-to-treat (ITT) analysis, and dropout rate of patients); and source of data (i.e., direct and indirect (from figures or calculated)). Then, all covariates were entered into a multivariate meta-regression model using a backward elimination approach with a removal criterion of P > 0.05. Additionally, continuous covariates were obtained from the meta-regression analyses to investigate whether relationships were linear and consistent with the results of the categorical analysis. The proportion of total between-study variances was explained by the models and reported as R^2^. We used meta-regression models to test between-subgroup interactions, and a P value ≤0.05 indicated a significant difference.

Subgroup analyses were performed according to the source of heterogeneity or using covariates if possible. Condition type was used as the primary variable for the subgroup analyses.

Sensitivity analyses were performed to identify trials that disproportionately contributed to the observed heterogeneity. This was accomplished using jack-knife analysis, omitting each study one by one to assess its impact on the summary estimate. Galbraith plots were used to conduct a visual inspection of possible outlier studies that had excessive influence on the overall estimate. Metatrim analysis was used to explore possible missing trials to verify the robustness of the results after these trials were added.

Publication bias was explored using a contour-enhanced funnel plot and Egger’s test if there were up to 10 eligible studies included in the meta-analysis.

All results were shown with 95% confidence intervals. All analyses were performed with STATA 12.0 software (StataCorp LP, College Station, TX).

### Best evidence synthesis

The clinical significance for the SMD was rated as small (<0.40), moderate (0.40 ~ 0.70) or large (>0.70) according to variation in Cohen’s interpretation of effect size^22^.

Based on the results of our systematic review, we used the GRADE system to rate the quality of the evidence^23^. The relative importance of each outcome was scored as critical to the decision (7–9), important but not critical to the decision (4–6), or not important to the decision (1–3). The quality of evidence for each outcome was scored as high, moderate, low, or very low (see Supplementary Tables S2 and S3). Although the evidence based on the included randomized controlled trials (RCTs) was initially rated as high quality, the quality could be downgraded based on the following five factors: study limitations, inconsistency, directness, preciseness, or reporting bias. Similarly, the quality could be upgraded based on three factors: large effect size, dose-response gradient, or plausible confounders that would have reduced the effect. Eventually, GRADEpro 3.6 software^24^ was used to compile and analyze the evidence.

## Results

### Literature search

**Figure 1:**
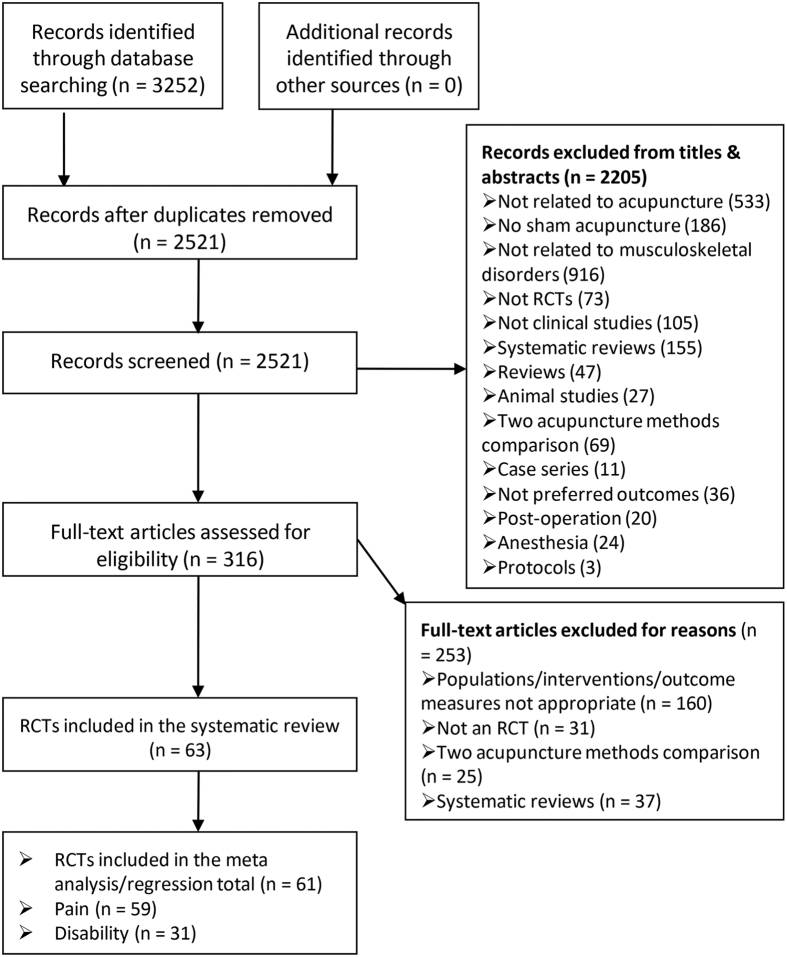
Flow chart.

Our search strategy identified 3252 potentially eligible articles (Fig. 1). A total of 731 duplicates were excluded, and 2205 additional records were also excluded based on their titles or abstracts for reasons such as not related to acupuncture or musculoskeletal disorders, not SA controlled, or not an RCT. After full-text articles were assessed for eligibility, 253 records were excluded for reasons such as irrelevance of the specified PICO (patient intervention comparison outcome), not an RCT, or in systematic review format. Eventually, 63 RCTs^25^,^26^,^27^,^28^,^29^,^30^,^31^,^32^,^33^,^34^,^35^,^36^,^37^,^38^,^39^,^40^,^41^,^42^,^43^,^44^,^45^,^46^,^47^,^48^,^49^,^50^,^51^,^52^,^53^,^54^,^55^,^56^,^57^,^58^,^59^,^60^,^61^,^62^,^63^,^64^,^65^,^66^,^67^,^68^,^69^,^70^,^71^,^72^,^73^,^74^,^75^,^76^,^77^,^78^,^79^,^80^,^81^,^82^,^83^,^84^,^85^,^86^,^87^ (6382 participants) were included in our systematic review. Of these, 61 (59 trials reporting pain, and 31 reporting disability) reported continuous data and performed a meta-analysis, and 2^40^,^42^ reported pain as dichotomous data. The latter were also subjected to qualitative analysis. Fifty-nine trials that reported pain as continuous data were also included in the meta-regression. The kappa value for the agreement between reviewers (QLY and JTM) was 0.91, which indicated excellent agreement.

### Study characteristics

All of the included studies were published between 1975 and 2013 (median 2007). The sample sizes ranged from 10 to 745 individuals (median 42, IQR 28 to 99, total 6382). Eight types of conditions were included: NP, SP, LBP, OA, RA, arm pain (AP), FM, and MP (Table 1). The basic characteristics of the included trials are shown in Supplementary Table S4; the demographic characteristics are shown in Supplementary Table S5; the acupuncture and SA characteristics are shown in Supplementary Tables S6 and S7; the reasons for trial exclusion are shown in Supplementary Table S8; and the data conversion and data extraction from figures are shown in Supplementary Table S9.

#### Participant characteristics

The proportions of females ranged from 0% to 100% (median 70.3%). Six studies included only women, one included only men, 53 included both women and men, and 3 did not report gender. The mean age ranged from 20.86 to 76.01 years (median 47.9), and all the participants were adults (age ≥18 years). Sixty-three studies reported the mean pain intensity at baseline, which ranged from 2.73 to 8.94 (median 6.05) on the VAS 10 cm. Four studies reported acute pain (<3 months, NP = 1, LBP = 3) from the duration of pain at baseline; others reported chronic pain (≥3 months).

#### Intervention characteristics

For all trials, there was a range of 1 to 24 treatment sessions (median 8, IQR 3.5 to 10); the total treatment periods ranged from 1 to 26 weeks (median 4, IQR 3 to 6); and the treatment frequencies ranged from 1 to 7 times/week (median 2, IQR 1 to 2). The most common treatment duration for each one-treatment session was 20 or 30 minutes. For some of the trials, the numbers of acupoints were not clearly reported, especially for individualized acupuncture groups in which the number of acupoints varied from patient to patient. Therefore, gross estimations were made on the basis of the descriptions included in the trial reports. The number of points ranged from 1 to 19 (median 9, IQR 4.7 to 12).

#### Sham acupuncture characteristics

Currently, SA is typically designed according to two factors: sham needle location (i.e., the same acupoints as real acupuncture, lateral to real acupoints, or acupoints of different or irrelevant conditions) and sham needle depth (i.e., non-penetration, superficial penetration, or normal penetration). After permutation and combination were calculated, eight SA types were identified. Twenty-five (39.7%) trials used a sham blunt needle with non-penetration at the same acupoints as in the intervention group.

### Risk of bias and methodological design

**Figure 2:**
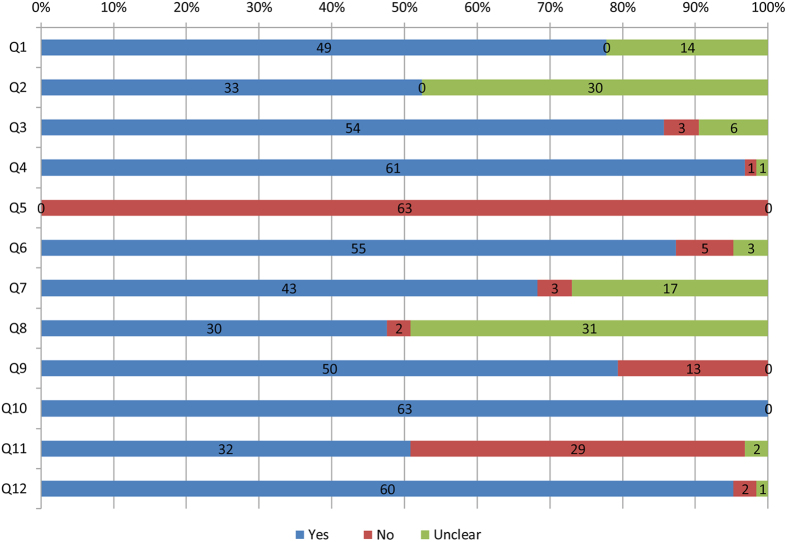
Risk of bias for the included studies. Q, question. Q1, Was the method of randomization adequate? Q2, Was the treatment allocation concealed? Q3, Were the groups similar at baseline regarding the most important prognostic indicators? Q4, Was the patient blinded to the intervention? Q5, Was the care provider blinded to the intervention? Q6, Was the outcome assessor blinded to the intervention? Q7, Were co-interventions avoided or similar? Was the compliance acceptable in all groups? Was the dropout rate described and acceptable? Was the timing of the outcome assessment similar in all groups? Was intention-to-treat analysis included? Are reports of the study free from suggestion of selective outcome reporting?

The quality scores of all of the studies ranged from 4 to 11 (median 8, IQR 6 to 9) (Supplementary Table S10, Fig. 2). Sixteen studies were of low quality (score ≤6), and the remaining 47 studies were of high quality (score >6). The dropout rates ranged from 0% to 33.3% (median 3.84%, IQR 0% to 14.6%); 50 studies reported less than 15% attrition. Thirty-two studies carried out ITT analyses, 29 did not, and 2 were unclear. Forty-nine studies reported their methods of randomization (computer or central call), and the other 14 trials were unclear. Thirty-three trials reported right allocation concealments (opaque seals and central call); the remaining 30 did not report this clearly. Fifty-five trials were double-blinded (patients and assessors were blinded); however, none of the studies had the caregivers blinded. Three of the included trials had a crossover design, while the others had a parallel design. One or more additional treatments, such as the use of non-steroidal anti-inflammatory drugs (NSAIDs), were added to both groups in many of the trials.

### Effects of acupuncture and meta-regression under different conditions

#### All conditions (overall summary effects)

After all of the trials were pooled, statistically significant differences in favor of the intervention group for both pain relief (59 trials, 4980 individuals, SMD −0.61, 95% CI, −0.76 to −0.47; P < 0.001) and disability improvement (31 trials, 4876 individuals, −0.77, −1.05 to −0.49; P < 0.001) were found and in both cases seemed to be of moderate to large clinical significance based on the variance of Cohen’s definitions. However, both cases showed significant heterogeneities (P < 0.001), with I^2^ values of 80.4% for pain and 94.7% for disability. Therefore, these analyses suggested that real acupuncture had a greater effect on pain relief and disability improvement than did SA. Forest plots (see Figures S1 and S2 in supplementary information file) were used to show the effect sizes, confidence intervals, and proportion weightings in both pain and disability for individual trials and for all the trials pooled. The largest weightings for any individual trial were 2.49% for pain and 3.72% for disability. The number of trials that showed significant differences favoring real acupuncture over SA were 30 (50.85%) for pain and 13 (41.94%) for disability. One trial^38^ (Goldman 2008) reported that SA was superior to real acupuncture for pain associated with lateral epicondylitis. The subgroup and sensitivity analyses were shown in Table 2 (for pain) and Table 3 (for disability). The results of the meta-regression and the possible sources of heterogeneities for each individual pain condition were also summarized (Tables 4 and 5).

#### Neck pain

**Figure 3:**
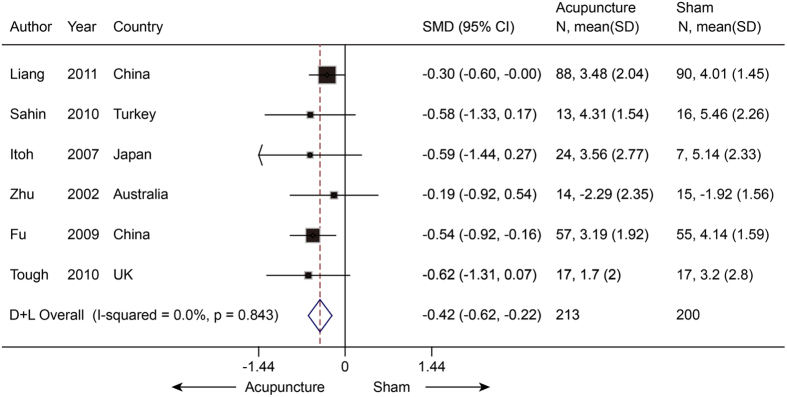
Meta-analysis of Acupuncture versus SA for NP in Pain. CI, confidence interval; NP, neck pain; SA, sham acupuncture; SD, standard deviation.

Six studies^25^,^26^,^27^,^28^,^29^,^30^ (n = 413) reported mean pain scores and were pooled with a moderate effect in favor of real acupuncture over SA (SMD −0.42, −0.62 to −0.22; P < 0.001) (Fig. 3) with no significant heterogeneity (I^2^ = 0%, P = 0.84).The jack-knife analysis did not change the results significantly. Egger’s test showed no evidence of publication bias (coefficient = −0.63; P = 0.39).

**Figure 4:**
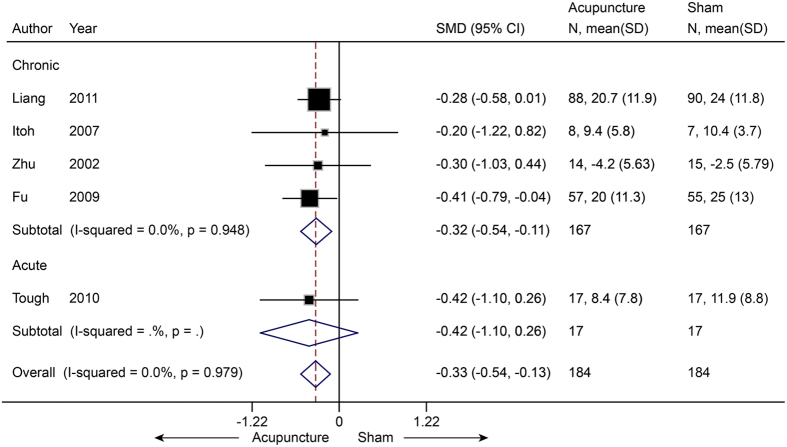
Meta-analysis of Acupuncture versus SA for NP in Disability. CI, confidence interval; NP, neck pain; SA, sham acupuncture; SD, standard deviation.

For disability, five studies^25^,^27^,^28^,^29^,^30^ (n = 368) were pooled, and the SMD was −0.33 (−0.54 to −0.13, P = 0.002) (Fig. 4). This result indicated that real acupuncture had a small effect on disability improvement compared to SA. No significant heterogeneity was found (I^2^ = 0%, P = 0.979). The jack-knife analysis did not change the results significantly. Egger’s test suggested no evidence of publication bias (coefficient = −0.01; P = 0.99).

#### Shoulder pain

**Figure 5:**
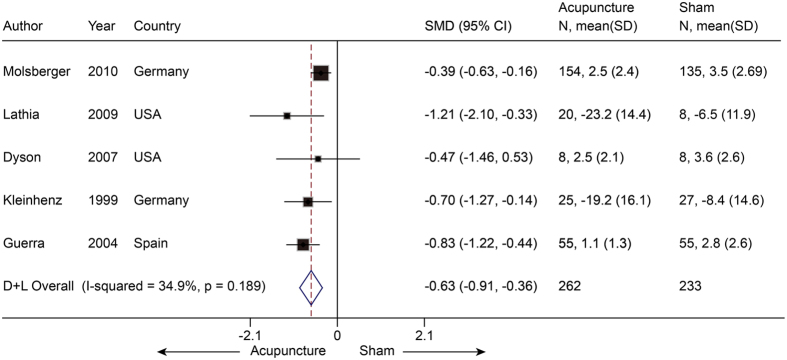
Meta-analysis of Acupuncture versus SA for SP in Pain. CI, confidence interval; SA, sham acupuncture; SP, shoulder pain; SD, standard deviation.

Five trials^31^,^32^,^33^,^34^,^35^ with a total of 495 participants compared mean pain scores between real acupuncture and SA. The SMD was −0.63 (−0.91 to −0.36, P < 0.001) (Fig. 5), indicating that there was a moderate effect favoring real acupuncture over SA. There was no evidence of significant heterogeneity (I^2^ = 34.9%, P = 0.19). The result was still robust after jack-knife analysis. No significant publication bias was found using Egger’s test (coefficient = −1.50; P = 0.23). We performed a meta-regression to explore the likely source of heterogeneity and found that sham needle location had an R^2^ of 100%, which indicated that this covariate could explain all the heterogeneity.

Two studies^32^,^34^ reported disability and were pooled (n = 129), with a SMD of −1.50 (−5.46 to 2.46). No significant difference was found between groups (P = 0.46). However, significant heterogeneity was shown (I^2^ = 96.1%, P < 0.001).

#### Neck pain and shoulder pain

Two studies^36^,^37^ (n = 58) reported pain intensity in patients with both NP and SP. We pooled the studies and found no significant difference between groups (SMD −0.52, −1.31 to 0.28; P = 0.20). The heterogeneity was not significant (I^2^ = 53.3%, P = 0.14).

**Figure 6:**
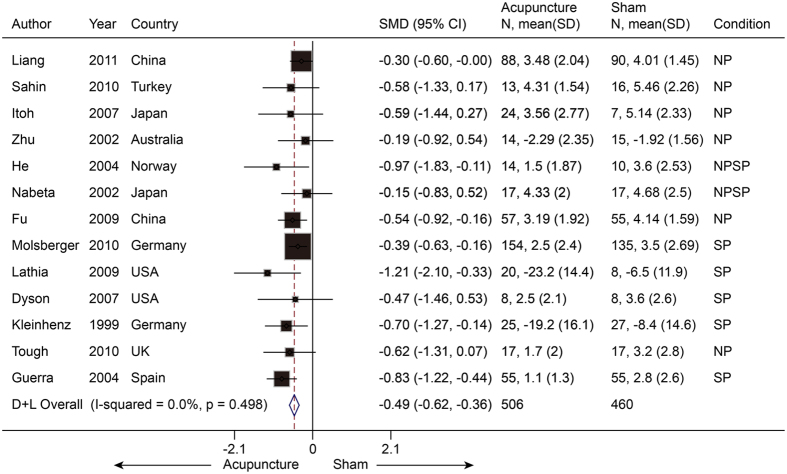
Meta-analysis of Acupuncture versus SA for NPSP in Pain. CI, confidence interval; NPSP, neck pain and shoulder pain; SA, sham acupuncture; SD, standard deviation.

**Figure 7:**
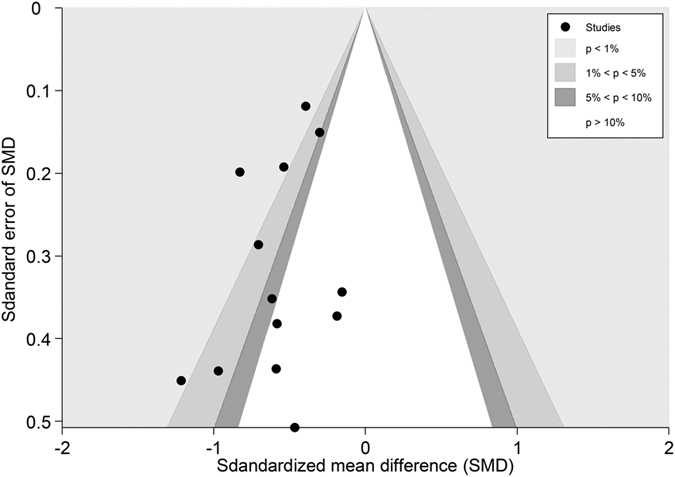
Contour-enhanced Funnel Plot of Acupuncture versus SA for NPSP in Pain. Visual inspection of the funnel plot suggested symmetry. Specifically, most of the trials had negative results (i.e., more trials in areas of statistical non-significance), indicating no evidence of publication bias.

We then pooled these two studies with the studies noted above that reported NP or SP, resulting in 13 trials (n = 966) with an SMD of −0.49 (−0.62 to −0.36, P < 0.001) (Fig. 6). This suggests that real acupuncture has a moderate effect on NP and SP compared to SA. All of the trials were statistically homogeneous (I^2^ = 0%, P = 0.47). The jack-knife analysis did not result in significant changes in the results. Both Egger’s test and the contour-enhanced funnel plot indicated no presence of publication bias (coefficient = −1.50; P = 0.23) (Fig. 7).

#### Low back pain

**Figure 8:**
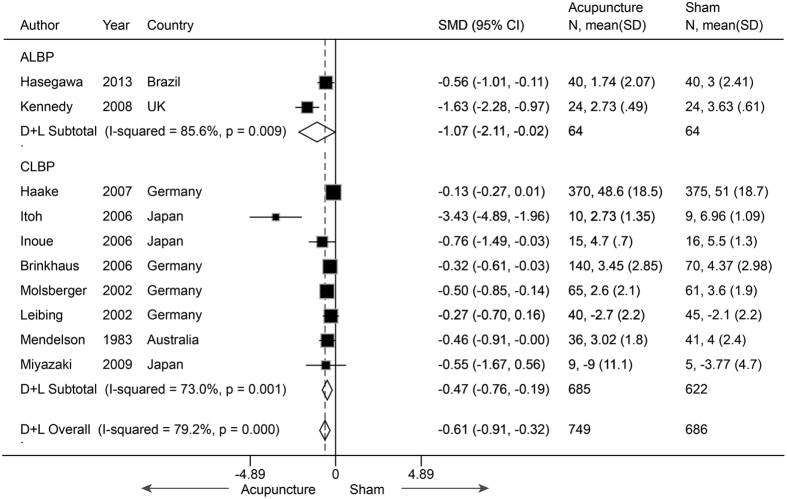
Meta-analysis of Acupuncture versus SA for LBP in Pain. CI, confidence interval; LBP, low back pain; SA, sham acupuncture; SD, standard deviation.

Ten studies^41^,^43^,^45^,^46^,^47^,^48^,^49^,^50^,^51^,^52^ (n = 1435) reported mean pain scores for LBP. The pooled SMD was −0.61 (−0.91 to −0.32, P < 0.001) (Fig. 8), which indicated a moderate effect favoring real acupuncture. However, the results were significantly heterogeneous (I^2^ = 79.2%, P < 0.001). The meta-regression identified sham needle depth (i.e., non-penetration, superficial penetration, or normal penetration) as the main source of the heterogeneity (R^2^ = 62.69%), explaining 62.69% of the heterogeneity. The pooled SMDs within the sham needle subgroups were −1.23 (−1.98 to −0.48) for non-penetration, −0.19 (−0.31 to −0.08) for superficial penetration and −0.50 (−0.85 to −0.14) for normal penetration. Publication bias was identified by Egger’s test (coefficient = −3.01; P = 0.003). Metatrim analysis found that two studies with positive effects favoring real acupuncture were missing. After these trials were filled, a larger effect was found (SMD −0.84, −1.26 to −0.42). A subgroup analysis was also performed according to condition duration (acute or chronic). Eight of these studies^43^,^46^,^47^,^48^,^49^,^50^,^51^,^52^ focused on chronic LBP, with a pooled SMD of −0.47 (−0.76 to −0.19, P = 0.001). This result indicated that real acupuncture was more effective than SA, but the effect decreased to moderate. The heterogeneity was still significant (I^2^ = 73.0%, P = 0.001), and sham needle depth was still the source of heterogeneity (R^2^ = 80.15%). The jack-knife analysis indicated that the results were robust. Egger’s test suggested publication bias (coefficient = −2.54; P = 0.01). Nevertheless, we conducted trim and fill analysis, and no study was filled. This indicated that the publication bias had a non-significant effect on the results. One study^47^ (Itoh 2006) with a smaller sample size (n = 19) but a very large effect size (SMD = −3.43) was found to be the source of heterogeneity based on the Galbraith plot. After removing this study, the result was still robust (SMD −0.30, −0.45 to −0.15, P < 0.001), and significant heterogeneity (I^2^ = 22.6%, P = 0.26) was not found, although publication bias was present (coefficient = −1.67; P = 0.01). Two of these ten trials^41^,^45^ reported on acute LBP, and both had a favorable result for real acupuncture. The pooled SMD was −1.07 (−2.11 to −0.02, P = 0.045). The heterogeneity was not significant (I^2^ = 22.6%, P = 0.26). In addition, one study^42^ reported on acute LBP with dichotomous data, and no significant difference was found between groups (OR 1.19, 0.62 to 2.28, P = 0.61).

**Figure 9:**
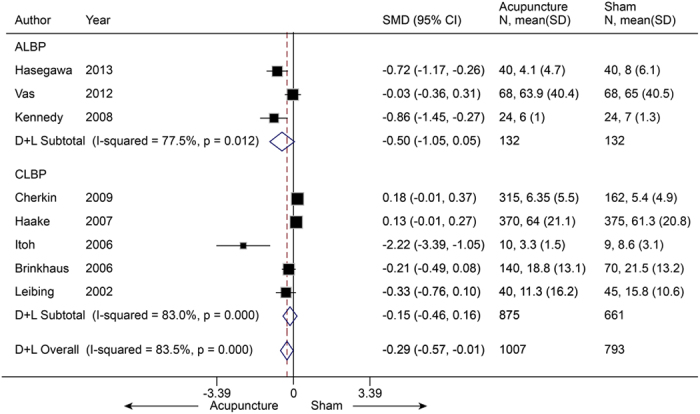
Meta-analysis of Acupuncture versus SA for LBP in Disability. CI, confidence interval; LBP, low back pain; SA, sham acupuncture; SD, standard deviation.

Eight trials^41^,^42^,^44^,^45^,^46^,^47^,^49^,^51^ (n = 1800) reported on disability in LBP, with a pooled SMD of −0.29 (−0.57 to −0.01, P = 0.04) (Fig. 9), which suggested that real acupuncture had a small effect compared to SA. However, heterogeneity was present (I^2^ = 83.5%, P < 0.001). The jack-knife analysis suggested the results changed significantly and removal of any one of the five individual trials could result in non-significance (P > 0.05). Five of these eight trials^44^,^46^,^47^,^49^,^51^ (n = 1536) reported disability in chronic LBP, and non-significant differences were found between groups (SMD −0.15, −0.46 to 0.16, P = 0.34). The results were heterogeneous across trials (I^2^ = 83%, P < 0.001). Egger’s test suggested a publication bias (coefficient = −4.18; P = 0.01). The contour-enhanced funnel plot showed an asymmetry due to the small-study effect. We adjusted this bias by removal of the small study^47^ (n = 19) (Itoh 2006), and the publication bias was eliminated (coefficient = −3.64; P = 0.11), the heterogeneity was lowered (I^2^ = 66%, P = 0.03), and the pooled SMD was 0.00 (−0.20 to 0.20). For acute LBP, the remaining three studies^41^,^42^,^45^ (n = 264) achieved a pooled SMD of −0.50 (−1.05 to 0.05, P = 0.07), which suggested no significant difference between groups. However, significant heterogeneity was still present (I^2^ = 77.5%, P = 0.01).

#### Osteoarthritis

**Figure 10:**
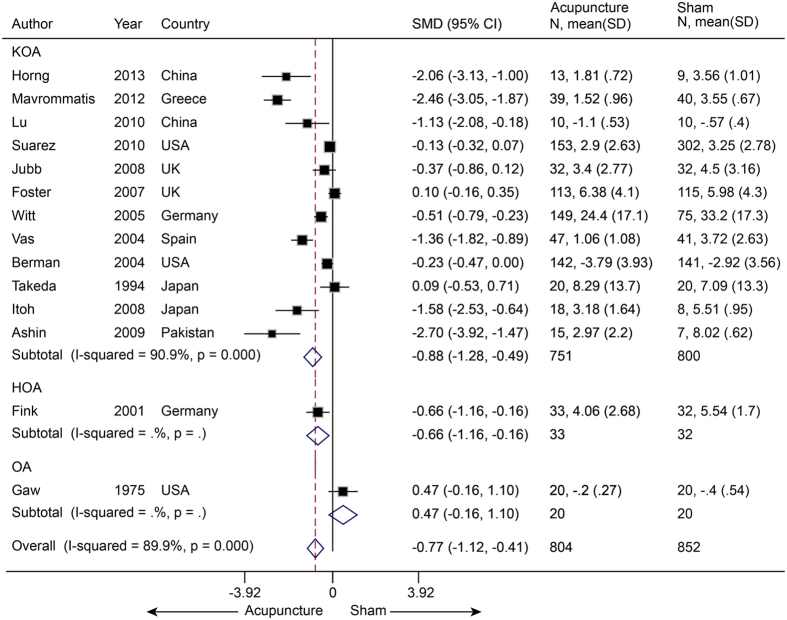
Meta-analysis of Acupuncture versus SA for OA in Pain. CI, confidence interval; OA, osteoarthritis; SA, sham acupuncture; SD, standard deviation.

**Figure 11:**
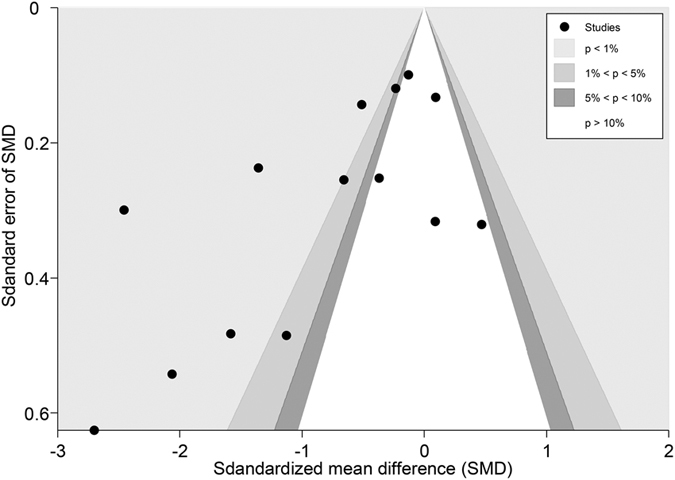
Contour-enhanced Funnel Plot of Acupuncture versus SA for OA in Pain. Visual inspection of the funnel plot suggested symmetry. Specifically, most trials had negative results (i.e., more trials in areas of statistical non-significance), indicating no evidence of publication bias.

Fourteen studies^53^,^54^,^55^,^56^,^57^,^58^,^59^,^60^,^62^,^63^,^64^,^65^,^66^,^67^ (n = 1656) reported pain in patients with osteoarthritis (1 hip OA^66^, 12 knee, 1 both^67^). The pooled SMD was −0.77 (−1.12 to −0.41, P < 0.001) (Fig. 10), which indicated that real acupuncture had a larger effect on OA pain than SA. The jack-knife analysis showed the results were robust and had no significant change. However, there was high heterogeneity (I^2^ = 89.9%, P < 0.001). Univariate meta-regression was used to evaluate the continents on which the studies took place, the publication years and the sample sizes, and we found that these factors could explain the heterogeneity with R^2^ values of 16.95%, 29.87% and 11.83%, respectively. Multivariate meta-regression indicated that these three covariates could explain the majority of the heterogeneity (R^2^ = 62.52%), suggesting that these covariates were the source of the heterogeneity. The contour-enhanced funnel plot suggested an asymmetry (Fig. 11), and Egger’s test indicated publication bias (coefficient = −3.71; P = 0.02). However, metatrim analysis found that no study was missing or should be added.

**Figure 12:**
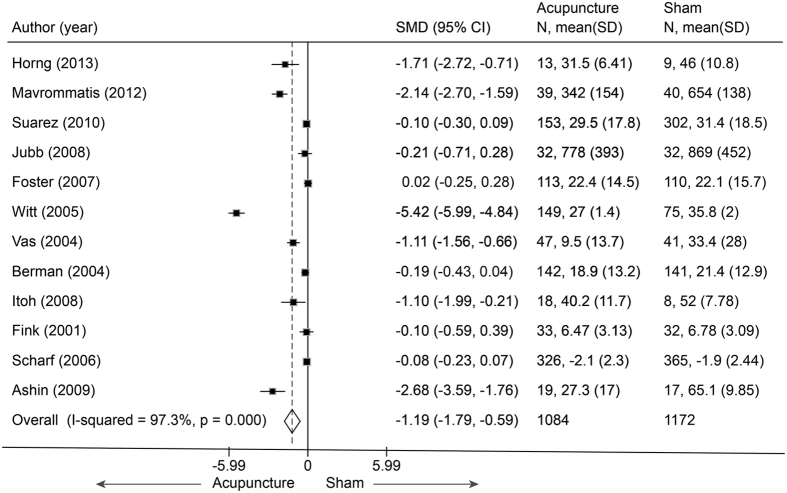
Meta-analysis of Acupuncture versus SA for OA in Disability. CI, confidence interval; OA, osteoarthritis; SA, sham acupuncture; SD, standard deviation.

**Figure 13:**
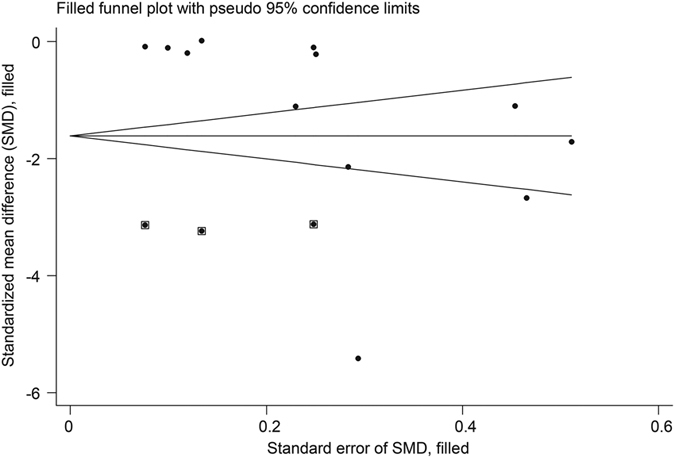
Metatrim Analysis of Acupuncture versus SA for OA in Pain. The dots in the squares were the studies filled. There were two trials with positive effects filled.

Twelve trials^53^,^54^,^55^,^57^,^58^,^59^,^60^,^61^,^62^,^63^,^64^,^66^ (n = 2256) reported on disability in OA (1 hip^66^, 11 knee) with a pooled SMD of −1.19 (−1.79 to −0.59, P < 0.001) (Fig. 12). This suggested that real acupuncture had a larger effect on individuals with OA than did SA. The jack-knife analysis found that the results did not change significantly on the removal of any individual study. However, a high heterogeneity was observed across these studies (I^2^ = 97.3%, P < 0.001). Univariate meta-regression indicated that sham needle location, pain at baseline (≥6 or <6) and an acupuncture-naive status (yes or unclear) had R^2^ values of 19.10%, 8.04% and 6.39%, respectively. We then assessed these three covariates using multivariate meta-regression and calculated a R^2^ of 51.68%, which indicated that these covariates could explain the majority of the heterogeneity. Asymmetry was observed in the contour-enhanced plot, and evidence of publication bias was found with Egger’s test (coefficient = −6.92; P = 0.03). Metatrim analysis indicated that three trials with positive effects were missing (Fig. 13). Adding these trials into the pooling yielded a larger benefit from real acupuncture, with a pooled SMD of −1.61 (−2.46 to −0.77).

#### Temporomandibular joint pain (myofascial pain)

**Figure 14:**
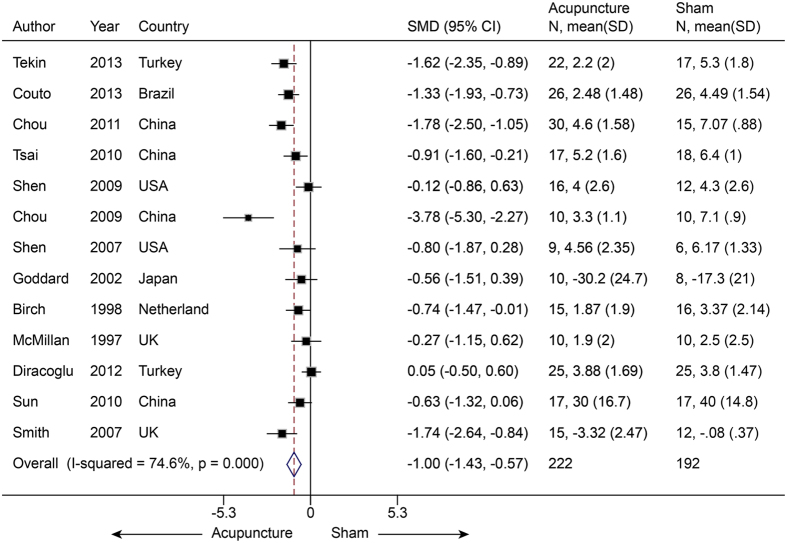
Meta-analysis of Acupuncture versus SA for MP in Pain. CI, confidence interval; MP, myofascial pain; SA, sham acupuncture; SD, standard deviation.

Thirteen studies^75^,^76^,^77^,^78^,^79^,^80^,^81^,^82^,^83^,^84^,^85^,^86^,^87^ (n = 414) were pooled to compare real acupuncture with SA in patients with MP. The real acupuncture showed a favorable effect on pain relief. The pooled SMD was −1.00 (−1.43 to −0.57, P < 0.001) (Fig. 14), with significant heterogeneity (I^2^ = 74.6%, P < 0.001). This result indicated that real acupuncture had a larger effect than SA. The removal of any one of the studies did not significantly affect the results, which had means ranging from −0.86 to −1.10 (P < 0.001) in the jack-knife analysis. We used univariate meta-regression to explore the likely source of heterogeneity, and two covariates (sham needle location and depth) were identified with R^2^ values of 46.46% and 47.20%, respectively. We then assessed these two covariates with multivariate meta-regression and calculated an R^2^ of 99.52%. This suggested that these covariates could explain 99.52% of the heterogeneity. Egger’s test did not suggest publication bias (coefficient = −1.50; P = −0.23). However, it should be noted that no studies reported disability scores.

#### Fibromyalgia

**Figure 15:**
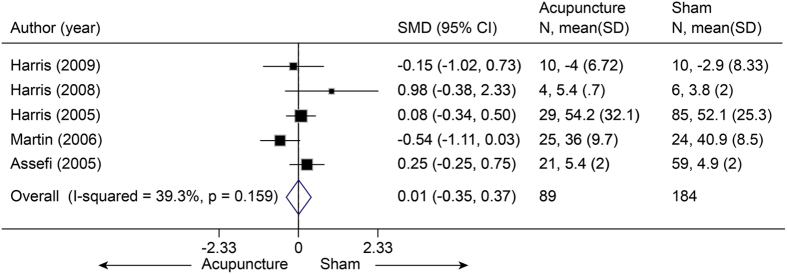
Meta-analysis of Acupuncture versus SA for FM in Pain. CI, confidence interval; FM, fibromyalgia; SA, sham acupuncture; SD, standard deviation.

Five studies^70^,^71^,^72^,^73^,^74^ (n = 631) were included for analysis of pain associated with FM. The pooled SMD was 0.01 (−0.35 to 0.37, P = 0.96) (Fig. 15), suggesting a non-significant difference between real acupuncture and SA. There was no evidence of significant heterogeneity (I^2^ = 39.3%, P = 0.16). Meta-regression indicated that sham needle depth could explain all of the heterogeneity (R^2^ = 100%). No evidence of publication bias was found using Egger’s test (coefficient = 0.75; P = −0.72). The jack-knife analysis indicated that the results did not change significantly.

Two studies^72^,^73^ (n = 163) were pooled for analysis of disability associated with FM, with a SMD of −0.38 (−0.72 to −0.05, P = 0.03). Non-significant heterogeneity was found (I^2^ = 0%, P = 0.35).

#### Lateral epicondylitis (tennis elbow or arm pain)

Two trials^38^,^39^ (n = 160) reported both pain and disability arising from lateral epicondylitis (tennis elbow), with non-significant SMDs of −0.18 (−1.33 to 0.97, P = 0.76) for pain and −1.63 (−5.37 to 2.11, P = 0.39) for disability. Both pain and disability had high heterogeneities, with I^2^ values of 90% and 98%, respectively. One trial^39^ (n = 118) (Fink 2002) showed a positive effect in favor of real acupuncture for both pain (SMD −0.80, −1.43 to −0.17) and disability (SMD −3.57, −4.56 to −2.58). However, another trial^38^ (Goldman 2008) (n = 42) reported that SA was superior to real acupuncture for pain relief (SMD 0.38, 0.01 to 0.74) and showed no difference for disability (SMD 0.25, −0.12 to 0.61). Additionally, one trial^40^ (n = 48) (Mosberger 1994) reported pain using dichotomous data, with an OR of 11.40 (2.95 to 44.00) favoring real acupuncture.

#### Rheumatic arthritis

Two studies^68^,^69^ (n = 76) investigated pain in patients with rheumatic disorders, with a SMD of −0.14 (−0.60 to 0.33, P = 0.57). No significant difference was found between the real and sham groups, and no statistical heterogeneity was observed (I^2^ = 0%, P = 0.32). Disability was not reported.

### Meta-regressions for exploring specific covariates for pain in overall conditions

Meta-regression of heterogeneity was possible only for the outcome of pain intensity, as it was our primary outcome measurement and was also more clinically relevant. The outcome of disability was reported in too few trials for the analysis to be robust and too few conditions for inclusive coverage of all the conditions. With regard to the number of SMDs used in each meta-regression, almost all the covariates were analyzed with 59 SMDs, but four of the covariates were excluded because some trials did not report data for these covariates (for example, one trial^65^ did not report data on age at baseline; therefore, only 58 SMDs were available for meta-regression analysis of age at baseline). These covariates were age at baseline (58 SMDs), pain at baseline (55 SMDs), proportion of females at baseline (56 SMDs), and sham needle depth (58 SMDs).

For univariate meta-regression of categorical covariates (Table 6), sample size of trial (<80 or ≥80) (R^2^ = 17.14%), year of publication (<2009 or ≥2009) (R^2^ = 10.48%), continent on which a trial was conducted (R^2^ = 6.79%), sham needle depth (R^2^ = 9.85%), sham needle location (R^2^ = 4.86%), and allocation concealment (R^2^ = 5.92%) appeared to be responsible for some of the heterogeneity in pain intensity. However, only three covariates (i.e., sample size of trial, year of publication, and continent) showed significant differences in interactions between subgroups (P < 0.05). Regarding trial sample size, the SMD for the smaller sample size (<80) was 0.53 lower than that for the larger sample size (≥80) (P = 0.01). Regarding year of publication, the SMD for the past five years (≥2009) was 0.50 lower than that for previous years (<2009) (P = 0.02). Finally, regarding continent on which the trial was conducted, the SMD for Asia was 0.37 lower than that for Europe and 0.73 lower than that for America (P = 0.04). Additionally, for the sham needle depth or location, even though these two covariates could explain some heterogeneities, no significant difference was found between subgroups via these covariates (both sham needle depth and location) (P for interactions were 0.09 for sham needle depth and 0.19 for sham needle location) (Table 6). Consequently, the SA type seemed to be not related to the estimated effect of real acupuncture.

**Figure 16:**
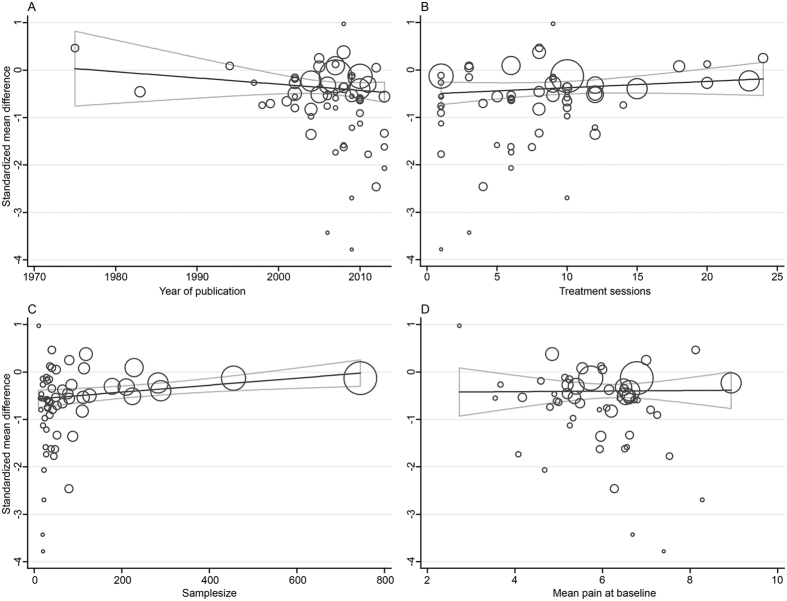
Meta-regression of Acupuncture versus SA for Overall Conditions in Pain (Part 1). CI, confidence interval; SA, sham acupuncture; SD, standard deviation.

**Figure 17:**
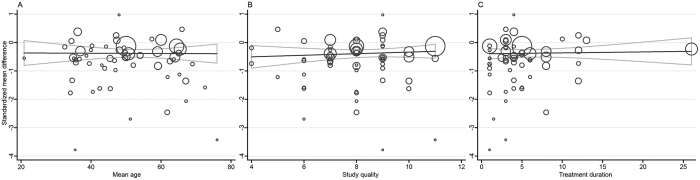
Meta-regression of Acupuncture versus SA for Overall Conditions in Pain (Part 2). CI, confidence interval; SA, sham acupuncture; SD, standard deviation.

We analyzed the strengths of the linear associations between the intervention effects (SMD) on pain intensity and each of the continuous study-level covariates (i.e., year of publication, mean age, mean pain at baseline, treatment session, treatment duration, study quality, sample size, and proportion of females). Year of publication explained 10.18% of the variation in effect sizes (P = 0.02): the SMD was an average of 0.03 lower for each 10-year increase in year of publication (coefficient = −0.033) (Fig. 16A). Treatment session explained 9.81% of the heterogeneity (P = 0.03): the SMD was 0.039 greater for each 1-treatment increase in treatment session (coefficient = 0.039) (Fig. 16B). However, this association was not significant across the sample sizes of trials (coefficient = 0.001, P = 0.054, R^2^ = 8.55%) (Fig. 16C). None of the other continuous covariates had a significant association with the sizes of the intervention effects (all P ≥ 0.17, R^2^ = 0.00%) (Fig. 17).

### Overall publication bias

**Figure 18:**
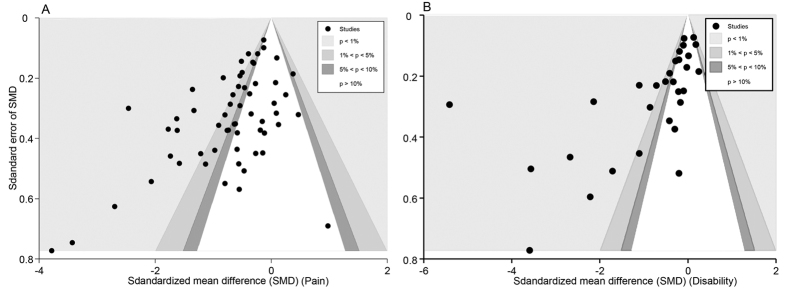
Contour-enhanced Funnel Plot of Acupuncture versus SA for Overall Conditions in Pain. Visual inspection of the funnel plot suggested symmetry. Specifically, most trials had negative results (i.e., more trials in areas of statistical non-significance), indicating no evidence of publication bias.

All the trials included in the meta-analyses were also included in the publication bias analyses (59 trials for pain, 31 trials for disability). For pain, the contour-enhanced funnel plot of the SMD showed a significant asymmetric scatter consistent with publication bias (Fig. 18A) (Egger’s test, coefficient = −2.23, P < 0.001). Nevertheless, we could not rule out the possibility of the small-study effect, as the asymmetry was attributable not only to three studies with small sample sizes and positive effects but also to one study^54^ (Mavrommatis 2012) with a larger sample size and a positive effect. We then performed metatrim analysis and found that three trials with positive effects were missing. After these three missing trials were filled, an even larger positive effect was found with a SMD of −0.68 (−0.84 to −0.53, P < 0.001). And these missing trials were likely to have had little effect on our findings, meaning that our result was still robust.

For disability, evidence of publication bias was also shown in the asymmetric contour-enhanced funnel plot (Fig. 18B) and in Egger’s test (coefficient = −4.79, P < 0.001). However, this bias could not be explained by the small-study effect because two larger studies^54^,^62^ (Witt 2005, Mavrommatis 2012) were also responsible for this bias. Metatrim analysis revealed that four trials with larger sample sizes and positive effects were missing; after these were filled, the difference favoring real acupuncture achieved an even greater positive effect with a SMD of −0.98 (−1.35 to −0.62, P < 0.001). This indicated that our results were still robust even with the presence of publication bias.

### Rating of the evidence

Eight types of musculoskeletal disorders were included in our review. As pain was the critical outcome measurement, the evidence was rated on the basis of pain. The levels of GRADE evidence and the reasons for upgrade and downgrade were shown (Table 7). The evidence quality for the overall conditions was rated as low because there were obvious heterogeneities (clinical and statistical) and publication biases. The levels of evidence quality were high for NP and SP; moderate for LBP, MP, and FM; low for OA; and very low for AP and RA.

## Discussion

### Key findings

Based on currently available evidence, our meta-analysis found that, overall, acupuncture was superior to SA in terms of pain relief and disability reduction for patients with musculoskeletal disorders. However, acupuncture was superior to SA for pain relief in only some of the individual conditions (chronic NP, SP, chronic LBP, OA, and MP). There were no differences between the groups for FM, AP, or RA, and we could not reach clear conclusion for acute NP, acute LBP, AP and RA for a small number of trials (≤2). For disability reduction, acupuncture was superior to SA in some conditions (chronic NP and OA), but there were no differences between groups for LBP, and we could not reach clear conclusion regarding acute NP, SP, FM, AP and MP for a few trials (≤2).

In a univariate meta-regression model, for individual conditions, sham needle location and/or depth could explain most or all of the heterogeneities for some conditions (SP, LBP, OA, MF, and FM), while other conditions were not applicable due to no heterogeneity (NP) or too few trials (RA and AP). For all conditions, a small portion of heterogeneity was explained by continent on which the study took place, year of publication, sample size, sham needle depth and location.

For sham needle depth or location, although these two covariates could explain some heterogeneity, no difference was found between subgroups via these covariates (both sham needle depth and sham needle location) (P for all interactions >0.05) (Tables 5 and 6). Consequently, SA type did not appear to be related to the estimated effect of real acupuncture.

We found a difference among the continent subgroups. The treatment effect in China was superior to that in other countries. The following speculations might account for this finding: acupuncture originated in China and was based on a set of relevant theories and practice experiences; and acupuncturists from China and adjacent countries usually had a five-year course of study. Additionally some other factors, such as psychological effect and publication bias, might also play a role in this difference.

The pooled SMD after 2009 was larger than it was before this date, which might have been the beneficial result of recent guidelines for quality control of acupuncture (STRICTA)^19^. This indicates that a good quality control of clinical acupuncture trial is needed.

### Design of sham acupuncture

Acupuncture causes both specific effects (real therapeutic effects) and non-specific effects (placebo effects). The factors influencing these specific effects include individual condition, type of pain, treatment duration and session number, selection of acupoints, needle apparatus, depth and angle of needle insertion, and quantity of stimulus^88^. The factors influencing the non-specific effects include patient responses to 1) being cared for and evaluated (i.e., the Hawthorne effect), 2) the use of placebo therapy, and 3) the physician-patient relationship^89^,^90^,^91^. The above theory may also be applicable to SA.

Klaus Linde *et al*.^92^ conducted a systematic review of 61 clinical trials to compare the efficacy of SA (19 trials) with those of other placebos (42 trials, including pharmacological and other physical placebos). The results showed that SA had a larger effect than other placebos. Thus, we speculated that so-called SA might have a specific effect beyond the placebo effect (i.e., a psychological effect). It was very difficult to evaluate the size of the specific effect of SA compared to that of real acupuncture. In addition, for each SA type applied, the psychological effects of real acupuncture and SA should be assessed individually in case a test was partial to either party.

Hence, the ideal SA must meet two primary criteria in clinical acupuncture trials: 1) the presence of no or only a small specific effect, thereby removing the influence on the evaluation of the acupuncture effect; and 2) no difference or high similarity between all other aspects to allow successful implementation of blinding.

SA needle depth involves either superficial penetration or non-penetration. In the former, the needle is inserted approximately 2 mm into the skin, while the latter uses a blunt needle that contacts the skin without penetrating it.

In the theory of traditional Chinese medicine, superficial penetration is a type of acupuncture that can be adopted to overcome the limitations imposed by some anatomical structures, such as the head, wrist, and ankle. Wu *et al*. found that superficial needling produced a good therapeutic effect for knee joint pain compared with routine acupuncture^93^. Likewise, superficial acupuncture was reported to be favorable for shoulder periarthritis by Lu and colleagues^94^. Additionally, Harris *et al*.^70^ found that superficial penetration stimulated specific regions of the brain and thereby had an analgesic effect. It is worth mentioning that, at the present time, the tissue layer or structure where acupuncture analgesia occurs and the functions of different tissue structures or layers in acupuncture analgesia remain unclear. It has been demonstrated that lightly touching the skin stimulates mechanoreceptors that are coupled to slow-conducting unmyelinated (C) afferents, resulting in activity in the insular region but not in the somatosensory cortex^95^. Activity in these C tactile afferents was deemed to induce a ‘limbic touch’ response, resulting in emotional and hormonal reactions. It is likely that control procedures in many acupuncture studies that were meant to be inert were in fact activating these C tactile afferents and, consequently, alleviating the affective component of pain^95^. Moreover, superficial acupuncture has yet to be strictly defined. Therefore, the decision to regard superficial acupuncture as a placebo is arbitrary.

The needling points used for non-penetration blunt-needle SA^96^ are different than those used in real acupuncture because the needles are not inserted into the skin, and there are no small hemorrhagic spots that may be detected by patients undergoing SA. This may also affect the implementation of patient blinding. For instance, individuals with more experience undergoing acupuncture therapy or greater knowledge about acupuncture were more likely to correctly guess the type of needle they received at ST36 compared to other points^97^. Thus, patients included in trials should be acupuncture-naïve; in other words, they should neither have knowledge of nor have received acupuncture treatment. In addition, acupoints should be selected at locations that patients cannot see.

Another type of SA uses needling points above 1.5 cm lateral to therapeutic acupoints and out of the meridian system while maintaining essentially the same manipulation technique and needle-insertion depth (approximately 10–20 mm) as real acupuncture^67^. This type of SA was designed according to the theory that sham acupoints have no therapeutic effect and that the meridian system is an effective factor. Controlled clinical trials have indicated that both acupoints and non-acupoints can produce therapeutic effects^67^,^98^. The possible mechanisms for this include changes in local circular and immune functions and the triggering of neural pathways that lead to diffuse noxious inhibitory controls^99^,^100^. A functional MRI study identified different reaction zones between acupoint needling and non-acupoint needling^101^, but there were considerable overlaps among the brain signals that arose in reaction to different acupoints. These findings seem to illustrate that the specificity of an acupoint is relative and that, even if the specificity of an acupoint really exists, the precise acupoint used is not that important for acupuncture’s effect.

Overall, many deficiencies exist in the currently available SA designs. The optimal type of SA design remains unclear. Future trials should compare different SA designs directly to provide more conclusive evidence regarding the optimal type of SA design.

### Comparison with other studies

Consistent with our current report, some previous systematic reviews have also found real acupuncture to be superior to SA for NP^102^, LBP^102^,^103^, OA^104^ and MP^105^. Two newly published meta-analyses^106^,^107^ found that real acupuncture had a more favorable effect than SA for LBP, with SMDs of −0.47^107^ and −0.58^106^. Our finding that real acupuncture was more effective than SA for NP and LBP was also verified by a more recent systematic review^102^.

We identified one trial^38^ (Goldman 2008) reporting that SA was superior to real acupuncture for pain associated with lateral epicondylitis. In the referenced trial, participants with persistent AP (N = 123) were randomly assigned to receive either real acupuncture or SA via 8 treatments over 4 weeks. A sham needle device (a blunt tip and retractable needle) was used. The reasons for the superiority of the SA device are not clear. One possibility is that the treatment effects were blunted in the real acupuncture group because of the higher rates of side effects, particularly mild pain during treatment. We speculate that this discomfort may have been due to the placement of needles in the arm that were in close proximity to the areas already experiencing pain.

Most side effects of acupuncture undergo spontaneous remission over several minutes or hours. Adverse reactions to acupuncture were rarely observed. Two prospective studies, with a total of 60,000 treatment sessions, did not find any serious side effects^108^,^109^. The total occurrence rate for meaningful minor side effects, including pain at acupuncture points, nausea and vomiting, and dizziness or syncope, was less than 0.1%.

### Strengths and weaknesses

A main strength of this study was its simultaneous assessment of acupuncture effectiveness (SA as the control group) in patients with almost all musculoskeletal disorders related to pain. This design provided a comprehensive review of the effects of acupuncture based on a registered number (CRD42014010760), using meta-regression analyses while considering possible sources of heterogeneity. Two independent reviewers extracted and analyzed the data and assessed the methodological quality. The majority of the studies were of high quality.

Moreover, our systematic review was conducted in strict accordance with the PRISMA statement^18^. The detailed characteristics of acupuncture or SA were extracted rigorously on the basis of the STRICTA statement^19^. Meta-regression was performed to explore possible sources of heterogeneity and to conduct indirect comparisons among subgroups. Metatrim analysis was conducted to sensitively assess publication bias. Furthermore, various statistical methods were employed according to Cochrane Handbook 5.1.0^110^ to convert existing data into available data, which eliminated possible selection bias. In particular, we conducted a meta-regression analysis of the characteristics of SA and found that differences in SA might not affect the evaluation of the effect size of acupuncture. At present, no other systematic review has used this approach.

The main weakness of this study was the relative paucity of high-quality RCTs. About half of the trials did not perform ITT analyses or correct allocation concealments. None of the studies blinded the caregivers because of the intrinsic characteristics of acupuncture. Furthermore, data on major clinical outcomes regarding pain for some conditions were available from only relatively few studies, especially for AP and RA (2 trials each). The small number of participating studies meant that the statistical power to detect differences was suboptimal. However, it remains possible that important differences exist in some conditions (i.e., NP, SP, LBP, OA, and MP). Moreover, the patients in many of the trials received additional treatments while undergoing acupuncture, such as NSAIDs as needed. Although these additional interventions were available in almost all parallel groups, they might have been unbalanced between groups, potentially minimizing the effect size of the outcome. Furthermore, the vast majority of the included studies did not report side effects or only reported equivocally, making it difficult to evaluate the side effects.

Although the subgroup and meta-regression analyses explained certain variations between studies, they could not explain all of them, and some variations were still unclear. Counter-enhanced funnel plots found small-study effects, which might have led to overrated effect sizes. On account of the relatively large number of a priori assumptions that were made, the reliability of the positive subgroup differences obtained should be lowered.

Finally, for patient-reported outcomes (e.g., pain and disability), patient expectations, preferences and satisfaction levels associated with treatment might have influenced the therapeutic effect or even acted as a dominant determinant^111^. However, almost none of the included studies evaluated and compared patient expectations between groups before or after acupuncture treatment.

### Future research and ongoing trials

Future studies should put the STRICTA statement into greater effect, such as when evaluating the qualification and experience levels of acupuncturists. Moreover, close attention should be paid to two points: 1) candidate patients’ expectations, preferences, and satisfaction levels associated with treatment should be taken into consideration^112^ and balanced between groups at baseline, and 2) acupuncture should be compared with other non-pharmaceutical therapies. Moreover, future systematic reviews should evaluate the effect of acupuncture compared with SA and the optimum design of SA for all pain-related disorders. Additionally, future studies should try to identify an ideal SA based on the influential factors of acupuncture and consider all of these factors comprehensively to minimize the specific effects of SA.

Careful monitoring by acupuncturists, including observation of treatments and frequent meetings to support them throughout a trial, is necessary to maintain a high degree of quality control^113^. Although numerous outcome measurements had been developed that were relevant to musculoskeletal pain care, whether these measures were appropriate for use by acupuncturists is still unclear. Further studies are warranted to explore whether established outcome measurements are useful for evaluating musculoskeletal pain following acupuncture, such as for chronic LBP^114^.

## Conclusion

Our review provided low-quality evidence that acupuncture has a moderate effect (approximately a 12-point pain reduction on the VAS 100 mm) on relieving pain associated with musculoskeletal disorders. Acupuncture was more effective than SA at relieving pain caused by chronic NP (high-level evidence), SP (high), chronic LBP (moderate), MP (moderate), and OA (low). There was no difference between groups for FM (moderate). There was not enough evidence for AP, RA, acute NP, and acute LBP. The type of SA used did not seem to be related to the estimated effect of real acupuncture.

## Additional Information

**How to cite this article**: Yuan, Q.-l. *et al*. Acupuncture for musculoskeletal pain: A meta-analysis and meta-regression of sham-controlled randomized clinical trials. *Sci. Rep.* **6**, 30675; doi: 10.1038/srep30675 (2016).

## Supplementary Material

Supplementary Information

## Figures and Tables

**Table 1:** Types of conditions included and overall characteristics.

Condition	Study (N)	Patients (N)	Treatment sessions (times)$	Period of treatment (weeks)$	Acupoint number$	Acupoints most frequently used*
NP & SP	13	966	9 (6, 10)	4 (3, 6)	8.3 (5.8, 12)	GB21, BL10, LI11, GB34
Neck	6	413	9 (6.8, 9)	4 (3, 5.5)	6 (5, 9)	GB21, GB20
Shoulder	5	379	10 (8, 12)	6 (5, 6)	10.5 (7.5, 12)	LI15, LI14, LI11, GB34
Both	2	58	3 or 10	3 or 3.5	6.5 or 14.5	
LBP	12	2098	7.8 (3.5, 11.5)	4 (3.3, 5)	12 (5.3, 15.5)	GB34, GB30, BL23, BL40
Acute	3	333	5 or 7.5	4 or 5	10.5 or 12	
Chronic	9	1765	9 (2.5, 12)	4 (2.5, 5.8)	13 (3, 16.3)	
OA	15	2347	7 (4.3, 10)	3 (3, 7.3)	10.3 (6, 12)	GB34, SP9, ST36, SP10
Knee	13	2242	9 (5.8, 10.5)	4 (3, 8)	10 (6, 11.8)	
Hip	1	65	3	1.5	12	
Both	1	40	1	1	NR	
AP	3	208	4 or 5	2	5 or 23	LI4
RA	2	76	10 or 20	5 or 10	12 or 23	ST36
FM	5	273	9 (9, 18)	4 (4, 12)	9 (9, 10.5)	LI4, SP6, ST36, LI11, GB34, GV20
MP	13	414	1 (1, 6)	1 (1, 3)	3 (2, 5.6)	LI4, TrP
Overall	63	6382	8 (3.5, 10)	4 (3, 6)	9 (4.7, 12)	GB34, LI4, ST36

NP, neck pain; SP, shoulder pain; LBP, low back pain; OA, osteoarthritis; RA, rheumatoid arthritis; AP, arm pain; FM, fibromyalgia; MP, myofascial pain.

^$^The results are shown as the median and interquartile range.

^*^The same acupoints were chosen in more than half (≥50%) of the studies that reported specific acupoints.

**Table 2:** Subgroup and sensitivity analysis (pain).

Conditions	Subgroup	Number of studies and participants	Effect size, standardized mean difference (95% CI)*	Effect size, P value	Heterogeneity, I^2^ (%)	Heterogeneity, P value
All conditions	Total	59, 4980	−0.61 (−0.76 to −0.47)	<0.001	80.3	<0.001
NP & SP						
	Subtotal	13, 966	−0.49 (−0.62 to −0.36)	<0.001	0	0.470
	NP					
	Subtotal	6, 413	−0.42 (−0.62 to −0.22)	<0.001	0	0.843
	Chronic	5, 379	−0.40 (−0.61 to −0.19)	<0.001	0	0.791
	Acute	1, 34	−0.62 (−1.31 to 0.07)	0.079	—	—
	SP					
	Subtotal	5, 495	−0.63 (−0.91 to −0.36)	<0.001	34.9	0.189
	Both					
	Subtotal	2, 58	−0.52 (−1.31 to 0.28)	0.20	53.3	0.143
LBP						
	Subtotal	10, 1435	−0.61 (−0.91 to −0.32)	<0.001	79.2	<0.001
		8, 1368#	−0.33 (−0.48 to −0.18)	<0.001	27.1	0.212
	Acute	2, 128	−1.07 (−2.11 to −0.02)	0.045	85.6	0.009
	Chronic	8, 1307	−0.47 (−0.76 to −0.19)	0.001	73.0	0.001
		7, 1288#	−0.30 (−0.45 to −0.15)	<0.001	22.6	0.257
OA						
	Subtotal	14, 1656	−0.77 (−1.12 to −0.41)	<0.001	89.9	<0.001
	Keen	12, 1551	−0.88 (−1.28 to −0.49)	<0.001	90.9	<0.001
	Hip	1, 65	−0.66 (−1.16 to −0.16)	0.01	—	—
	Both	1, 40	0.47 (−0.16 to 1.10)	0.14	—	—
MP						
	Subtotal	13, 414	−1.00 (−1.43 to −0.57)	<0.001	74.6	<0.001
FM						
	Subtotal	5, 273	0.01 (−0.35 to 0.37)	0.957	39.3	0.159
AP						
	Subtotal	2, 160	−0.18 (−1.33 to 0.97)	0.758	90.0	0.002
RA						
	Subtotal	2, 76	−0.14 (−0.60 to 0.33)	0.568	0	0.324

NP, neck pain; SP, shoulder pain; LBP, low back pain; OA, osteoarthritis; RA, rheumatoid arthritis; AP, arm pain; FM, fibromyalgia; MP, myofascial pain.

^*^Standardized mean difference (SMD) obtained using conventional random-effects model.

^#^Sensitivity analysis after some studies were dropped out.

**Table 3:** Subgroup and sensitivity analysis (disability).

Conditions	Subgroup	Number of studies and participants	Effect size, standardized mean difference (95% CI)*	Effect size, P value	Heterogeneity, I^2^ (%)	Heterogeneity, P value
All conditions	Total	31, 4876	−0.77 (−1.05 to −0.49)	<0.001	94.7	<0.001
NP						
	Subtotal	5, 368	−0.33 (−0.54 to −0.13)	0.002	0	0.979
	Chronic	4, 334	−0.32 (−0.54 to −0.11)	0.010	0	0.948
	Acute	1, 34	−0.42 (−1.10 to 0.26)	0.225	—	—
SP						
	Subtotal	2, 129	−1.50 (−5.46 to 2.46)	0.457	96.1	0.000
LBP						
	Subtotal	8, 1800	−0.29 (−0.57 to −0.01)	0.041	83.5	0.000
		7, 1462#	−0.18 (−0.42 to 0.06)	0.135	79.0	0.000
	Acute	3, 264	−0.50 (−1.05 to 0.05)	0.074	77.5	0.012
	Chronic	5, 1536	−0.15 (−0.46 to 0.16)	0.336	83.0	0.000
		4, 1517#	−0.00 (−0.20 to 0.20)	0.971	66.0	0.032
OA						
	Subtotal	12, 2256	−1.19 (−1.79 to −0.59)	<0.001	97.3	<0.001
	Keen	11, 2191	−1.29 (−1.93 to −0.65)	<0.001	97.6	<0.001
	Hip	1, 65	−0.10 (−1.79 to 0.39)	0.69	—	—
FM						
	Subtotal	2, 163	−0.38 (−0.72 to −0.05)	0.027	0	0.347
AP						
	Subtotal	2, 160	−1.63 (−5.37 to 2.11)	0.39	98.0	<0.001

NP, neck pain; SP, shoulder pain; LBP, low back pain; OA, osteoarthritis; RA, rheumatoid arthritis; AP, arm pain; FM, fibromyalgia; MP, myofascial pain.

^*^Standardized mean difference (SMD) obtained using conventional random-effects model.

^#^Sensitivity analysis after some studies were drop out.

**Table 4:** Results of the meta-regression and possible sources of heterogeneity for each individual condition related to pain.

Conditions	Number of studies and subjects	Heterogeneity, I^2^ (%)	Covariates	Adjusted R^2^ (%)$
NP & SP	13, 966	0	n.a.	n.a.
NP	6, 413	0	n.a.	n.a.
SP	5, 495	34.9	location of SN	100
LBP	10, 1435	79.2	depth of SN	62.69
OA	14, 1656	89.9	continents, years of publication, sample size	62.52
MP	13, 414	74.6	location of SN, depth of SN	99.52
FM	5, 273	39.3	depth of SN	100
AP	2, 160	90.0	n.a.	n.a.
RA	2, 76	0	n.a.	n.a.

SN, sham needle; NP, neck pain; SP, shoulder pain; LBP, low back pain; OA, osteoarthritis; RA, rheumatoid arthritis; AP, arm pain; FM, fibromyalgia; MP, myofascial pain; n.a., not applicable.

^$^Heterogeneity explained by covariate (%).

**Table 5:** Subgroup analysis of possible sources of heterogeneity explained by the design of sham acupuncture for individual conditions.

Covariate and classification (condition)	No. of studies or subsets	SMD (95% CI)*	P for SMD	P for heterogeneity†	I^2^ (%)	P for interaction‡	Adjusted R^2^$
Sham location (SP)							
Same	3	−0.84 (−1.14 to −0.54)	<0.001	0.633	0	0.46	100
Lateral	1	−0.47 (−1.46 to 0.53)	0.359	n.a.	n.a.		
Irrelevant	1	−0.39 (−0.63 to −0.16)	0.001	n.a.	n.a.		
Sham depth (LBP)							
Non-penetration	5	−1.23 (−1.98 to −0.48)	0.001	0.001	78.7	0.30	62.69
Superficial	4	−0.20 (−0.31 to −0.08)	0.001	0.404	0		
Normal	1	−0.50 (−0.85 to −0.14)	0.006	n.a.	n.a.		
Sham location (MP)							
Same	8	−1.37 (−1.88 to −0.86)	<0.001	0.001	70.4	0.46	46.46
Lateral	4	−0.19 (−0.57 to −0.19)	0.318	0.464	0		
Irrelevant	1	−0.74 (−1.47 to −0.01)	0.047	n.a.	n.a.		
Sham depth (MP)							
Non-penetration	7	−1.48 (−2.11 to −0.84)	<0.001	0.001	74.9	0.11	47.20
Superficial	6	−0.47 (−0.79 to −0.15)	0.004	0.320	14.7		
Normal	0						
Sham depth (FM)							
Non-penetration	3	−0.10 (−0.83 to 0.64)	0.790	0.124	52.1	0.51	100
Superficial	0						
Normal	2	0.15 (−0.17 to 0.47)	0.365	0.601	0		

FM, fibromyalgia; LBP, low back pain; MP, myofascial pain; SP, shoulder pain.

^*^Standardized mean difference (SMD) obtained using conventional random-effects model.

^$^Percentage of heterogeneity explained by covariate (%).

^†^P values for heterogeneity across studies computed using Cochrane’s Q test.

^‡^P values for comparisons between subgroups computed using χ^2^ test.

**Table 6:** Univariate meta-regression analysis of heterogeneity on effect of real acupuncture versus placebo needle acupuncture on pain immediately after the end of the intervention.

Covariate and classification	No. of studies or subsets	SMD (95% CI)*	P for SMD	P for heterogeneity†	I^2^ (%)	P for interaction‡	Adjusted R^2^$
Total	59	−0.61 (−0.76 to −0.47)	<0.001	<0.001	80.3	—	—
Sex							
Female (≥80%)	21	−0.70 (−0.88 to −0.52)	<0.001	<0.001	70.0	0.32	0.00
Both	35	−0.53 (−0.81 to −0.25)	<0.001	<0.001	83.2		
NR	3	−0.07 (−0.73 to 0.59)	0.83	0.02	74.0		
Age§							
<40	15	−0.65 (−0.99 to −0.31)	<0.001	<0.001	78.9	0.79	0.00
40–60	27	−0.51 (−0.69 to −0.33)	<0.001	<0.001	71.1		
≥60	16	−0.85 (−1.19 to −0.50)	<0.001	<0.001	88.8		
NR	1	0.09 (−0.53 to 0.71)	0.78	—	—		
Pain§							
≥6	27	−0.78 (−1.00 to −0.55)	<0.001	<0.001	85.0	0.19	0.00
<6	28	−0.51 (−0.72 to −0.30)	<0.001	<0.001	72.1		
NR	4	−0.19 (−0.68 to −0.31)	0.46	0.05	62.2		
Sample size							
<80	42	−0.83 (−1.06 to −0.59)	<0.001	<0.001	75.8	0.01	17.14
≥80	17	−0.30 (−0.45 to −0.15)	<0.001	<0.001	77.1		
Continent							
Asia	18	−1.00 (−1.37 to −0.63)	<0.001	<0.001	78.9	0.04	6.79
Europe	26	−0.63 (−0.83 to −0.42)	<0.001	<0.001	84.1		
America	15	−0.27 (−0.49 to −0.05)	0.017	0.001	61.8		
Year of publication							
≥2009	22	−0.94 (−1.23 to −0.64)	<0.001	<0.001	84.7	0.02	10.48
<2009	37	−0.44 (−0.61 to −0.28)	<0.001	<0.001	74.8		
Intervention stimulation							
Electricity	15	−0.83 (−1.18 to −0.49)	<0.001	<0.001	86.7	0.26	1.41
No electricity	44	−0.54 (−0.70 to −0.38)	<0.001	<0.001	76.6		
Sham stimulation∮							
Yes	9	−0.59 (−1.02 to −0.17)	0.006	<0.001	74.4	0.78	0.00
No	45	−0.64 (−0.80 to −0.47)	<0.001	<0.001	81.9		
NR	5	−0.39 (−0.81 to 0.03)	0.07	0.17	43.0		
Sham location¶							
Same	33	−0.79 (−1.04 to −0.54)	<0.001	<0.001	84.1	0.19	4.86
Lateral	16	−0.30 (−0.48 to −0.13)	0.001	0.088	34.3		
Irrelevant	10	−0.50 (−0.77 to −0.24)	<0.001	<0.001	80.8		
Sham depth∮							
Non-penetration	28	−0.90 (−1.21 to −0.60)	<0.001	<0.001	86.6	0.09	9.85
Superficial	20	−0.34 (−0.48 to −0.21)	<0.001	0.007	49.6		
Normal	10	−0.54 (−0.89 to −0.19)	0.003	0.001	68.8		
Licensed Acupuncturist							
Yes	44	−0.66 (−0.84 to −0.49)	<0.001	<0.001	82.4	0.44	0.00
NR	15	−0.48 (−0.74 to −0.21)	<0.001	<0.001	70.9		
Study quality							
≤6	11	−0.82 (−1.31 to −0.33)	<0.001	0.001	77.0	0.47	0.00
>6	48	−0.58 (−0.73 to −0.43)	<0.001	<0.001	81.0		
Allocation concealment							
Yes	49	−0.66 (−0.82 to −0.50)	<0.001	<0.001	81.2	0.11	5.92
No	2	−1.16 (-2.16 to −0.15)	0.02	0.06	72.6		
Unclear	8	−0.11 (−0.47 to 0.25)	0.56	0.04	51.4		
Intention to treat (ITT)							
Yes	24	−0.66 (−0.91 to −0.42)	<0.001	<0.001	74.6	0.80	0.00
No	33	−0.61 (−0.80 to −0.41)	<0.001	<0.001	83.8		
Unclear	2	−0.30 (−1.11 to 0.51)	0.47	0.09	65.2		
Blind survey							
Yes	18	−0.42 (−0.66 to −0.19)	<0.001	<0.001	75.7	0.30	2.05
Unclear	41	−0.69 (−0.88 to −0.51)	<0.001	<0.001	80.7		
Acupuncture naive patients							
Yes	32	−0.55 (−0.74 to −0.36)	<0.001	<0.001	82.4	0.58	0.00
No	2	−0.44 (−1.03 to 0.15)	0.15	0.23	29.6		
Unclear	25	−0.74 (−0.99 to −0.49)	<0.001	<0.001	78.6		
Continuous data							
Year of publication	59	−0.61 (−0.76 to −0.47)	<0.001	<0.001	80.3	0.02	10.18
Age§	58∌	−0.63 (−0.77 to −0.48)	<0.001	<0.001	80.4	0.41	0.00
Pain§	55∌	−0.64 (−0.80 to −0.49)	<0.001	<0.001	80.3	0.17	0.00
Number of points	53∌	−0.67 (−0.82 to −0.51)	<0.01	<0.001	80.6	0.05	7.10
Treatment session	59	−0.61 (−0.76 to −0.47)	<0.001	<0.001	80.3	0.03	9.81
Treatment duration	59	−0.61 (−0.76 to −0.47)	<0.001	<0.001	80.3	0.28	0.00
Study quality	59	−0.61 (−0.76 to −0.47)	<0.001	<0.001	80.3	0.99	0.00
Sample size	59	−0.61 (−0.76 to −0.47)	<0.001	<0.001	80.3	0.054	8.59
Proportion of females	56∌	−0.64 (−0.79 to −0.49)	<0.001	<0.001	79.7	1.00	0.00

NR, not reported.

^*^Standardized mean difference (SMD) obtained using conventional random-effects model.

^†^P values for heterogeneity across studies computed using Cochrane’s Q test.

^‡^P values for comparisons between subgroups computed using χ^2^ test.

^$^Percentage of heterogeneity explained by covariate (%).

^§^Mean baseline values for experimental (real acupuncture) and control (sham) arms of trials, explored with univariate meta-regression. Pain was determined based on the VAS 10 cm.

^¶^Assefi 2005 was excluded because three types of sham were used but not reported and we could not obtain the data of the subsets in the study. All types of sham in Harris 2005 achieved penetration but at different needle locations.

^∮^Assefi 2005 was excluded for the same reason as noted above, and Harris 2005 used and reported three types of sham. We obtained two subsets (same, different) for the covariate of sham needle location.

^∌^The actual number that reported related data.

NR, not reported.

**Table 7:** Rating of evidence for musculoskeletal pain.

Condition	No. of participants (studies)	Design	Limitations	Inconsistency	Indirectness	Imprecision	Other considerations	SMD (95% CI)	Quality†
All	4980 (59)	RCT	no serious^1^	serious^2^	no serious^3^	no serious^4^	publication bias^5^	−0.61 (−0.76 to −0.47)	Low
NP	413 (6)	RCT	no serious^1^	no serious^6^	no serious^3^	no serious^4^	none	−0.42 (−0.62 to −0.22)	High
SP	495 (5)	RCT	no serious^1^	no serious^6^	no serious^3^	no serious^4^	none	−0.63 (−0.91 to −0.36)	High
LBP	1435 (10)	RCT	no serious^1^	no serious^7^	no serious^3^	no serious^4^	publication bias^5^	−0.61 (−0.91 to −0.32)	Moderate
OA	1656 (14)	RCT	no serious^1^	serious^8^	no serious^3^	no serious^4^	publication bias^5^	−0.77 (−1.12 to −0.41)	Low
MP	414 (14)	RCT	no serious^1^	serious^8^	no serious^3^	no serious^4^	none	−1.00 (−1.43 to −0.57)	Moderate
FM	273 (5)	RCT	no serious^1^	no serious^6^	no serious^3^	serious^9^	none	0.01 (−0.35 to 0.37)	Moderate
AP	160 (2)	RCT	no serious^1^	serious^8^	no serious^3^	serious^9^	reporting bias^10^	−0.18 (−1.33 to 0.97)	Very low
RA	76 (2)	RCT	no serious^1^	no serious^6^	no serious^3^	serious^9^	reporting bias^10^	−0.14 (−0.60 to 0.33)	Very low

Notes: CI, confidence interval; SMD, standard mean difference; RCT, randomized controlled trial; NP, neck pain; SP, shoulder pain; LBP, low back pain; OA, osteoarthritis; MP, myofascial pain; FM, fibromyalgia; AP, arm pain; RA, rheumatic arthritis.

^†^Grades of evidence. High quality: further research is very unlikely to change our confidence in the estimate of effect; moderate quality: further research is likely to have an important impact on our confidence in the estimate of effect and may change the estimate; low quality: further research is very likely to have an important impact on our confidence in the estimate of effect and is likely to change the estimate; very low quality: we are very uncertain about the estimate.

^1^No serious limitations: the mean of the quality scores of all the studies for every condition was greater than 6 points, which indicated the quality of the studies was high, and all trials were RCTs. Sensitivity analysis excluding the trials with a high risk of bias did not change the results, so the evidence was not downgraded.

^2^Serious inconsistency: high statistical heterogeneity (I^2^ = 80.3%) not explained by subgroup analysis.

^3^Pain intensity was directly associated with clinical outcome.

^4^No serious imprecision: the effect size (SMD) was significantly different (P > 0.05).

^5^Publication biases were found to be significant (P < 0.05).

^6^No serious inconsistencies: no statistically significant heterogeneities were found (P > 0.05).

^7^No serious inconsistencies: although there was substantial heterogeneity across all related trials (I^2^ = 79.2%), our sensitivity analysis found that the heterogeneity was low with no statistical significance (I^2^ = 27.1%, P = 0.21), so the evidence was not downgraded.

^8^Serious inconsistency: high statistical heterogeneity (I^2^ > 70%).

^9^Serious imprecision: 95% CI crosses no treatment effect (SMD = 0).

^10^Reporting biases: only two trials with small sample sizes were found, so the evidence was downgraded.
