# Supplementary material for: Acupuncture for musculoskeletal pain: A meta-analysis and meta-regression of sham-controlled randomized clinical trials
Source: Sci Rep. 2016 Jul 29;6:30675. doi: 10.1038/srep30675 (PMC4965798; doi:10.1038/srep30675)

Acupuncture for musculoskeletal pain: A meta-analysis and meta-regression of sham-controlled randomized clinical trials

Authors:

Qi-ling Yuan, Peng Wang, Liang Liu, Fu Sun, Yong-song Cai, Wen-tao Wu, Mao-lin Ye, Jiang-tao Ma, Bang-bang Xu & Yin-gang Zhang

Search Strategy in Pubmed

#1 Conditions: (((("musculoskeletal abnormalities"[MeSH Terms]) OR "musculoskeletal diseases"[MeSH Terms])) OR ((((((((((((((joint[Title/Abstract]) OR hand[Title/Abstract]) OR wrist[Title/Abstract]) OR shoulder[Title/Abstract]) OR back[Title/Abstract]) OR spine[Title/Abstract]) OR lumbar[Title/Abstract]) OR neck[Title/Abstract]) OR cervical[Title/Abstract]) OR hip[Title/Abstract]) OR knee[Title/Abstract]) OR arm[Title/Abstract]) OR leg[Title/Abstract]) OR limb[Title/Abstract]) OR jaw[Title/Abstract])) OR (((((((((((((((((((((((((((((((((((rotator cuff tendinitis) OR ankylosing spondylitis) OR fibromyalgia) OR carpal tunnel syndrome) OR joint disorders) OR osteoarthritis) OR arthritis) OR rheumatoid arthritis) OR elbow pain) OR back pain) OR neck pain) OR neck disorder) OR shoulder pain) OR knee pain) OR tennis elbow) OR lateral elbow tendinopathy) OR subacromial bursitis) OR lateral epicondylitis) OR myofascial pain) OR heel pain) OR epicondyalgia) OR ankle distorsions) OR rheumatic disorders) OR tendinitis) OR rotator cuff lesions) OR tempromandibular pain) OR tempromandibular dysfunction) OR hemiplegic shoulder) OR contracture) OR knee extensor mechanism disorders) OR myalgia) OR arthralgia) OR gonarthrosis) OR extremity) OR extremities)

#2 Acupuncture: ((((((((((((acupuncture[MeSH Terms]) OR "acupuncture therapy"[MeSH Terms]) OR "acupuncture points"[MeSH Terms]) OR body acupuncture) OR electroacupuncture[MeSH Terms]) OR electro-acupuncture) OR electrical acupuncture) OR ear acupuncture[MeSH Terms]) OR auricular acupuncture) OR scalp acupuncture) OR dry needle) OR dry needling) OR triggers point) OR acupoint injection

#3 Study Design: ((((((((((((((random[Title/Abstract]) OR random[MeSH Subheading]) OR randomized controlled trial[Publication Type]) OR double blind method) OR single blind method) OR placebos) OR clinical trial[Publication Type]) OR clinical trials) OR controlled clinical trial[Publication Type])) OR ((clin*[Title/Abstract]) AND trial*[Title/Abstract])) OR ((((((singl*[Title/Abstract]) OR doubl*[Title/Abstract]) OR trebl*[Title/Abstract]) OR tripl*[Title/Abstract])) AND ((blind*[Title/Abstract]) OR mask*[Title/Abstract]))) OR placebo*[Title/Abstract]) OR random*[Title/Abstract])) OR random[MeSH Terms]

#4: 1 AND 2 AND 3

Table S1. Updated Method Guidelines for Systematic Reviews in the Cochrane Collaboration Back Review Group: A 12-Item Tool.

**Question**	**Item**	**Rating**
**Q1**	Was the method of randomization adequate?	Yes / No / Unsure
**Q2**	Was the treatment allocation concealed?	Yes / No / Unsure
**Q3**	Were the groups similar at baseline regarding the most important prognostic indicators?	Yes / No / Unsure
**Q4**	Was the patient blinded to the intervention?	Yes / No / Unsure
**Q5**	Was the care provider blinded to the intervention?	Yes / No / Unsure
**Q6**	Was the outcome assessor blinded to the intervention?	Yes / No / Unsure
**Q7**	Were co-interventions avoided or similar?	Yes / No / Unsure
**Q8**	Was the compliance acceptable in all groups?	Yes / No / Unsure
**Q9**	Was the drop-out rate described and acceptable?	Yes / No / Unsure
**Q10**	Was the timing of the outcome assessment in all groups similar?	Yes / No / Unsure
**Q11**	Did the analysis include an intention-to-treat analysis?	Yes / No / Unsure
**Q12**	Are reports of the study free of suggestion of selective outcome reporting?	Yes / No / Unsure

Table S2. Significance of the four levels of evidence

Quality level	Definition
High	We are very confident that the true effect lies close to that of the estimate of the effect
Moderate	We are moderately confident in the effect estimate: The true effect is likely to be close to the estimate of the effect, but there is a possibility that it is substantially different
Low	Our confidence in the effect estimate is limited: The true effect may be substantially different from the estimate of the effect
Very low	We have very little confidence in the effect estimate: The true effect is likely to be substantially different from the estimate of effect

Table S3. A Summary of the GRADE's Approach to Rating Quality of Evidence.

<table>
<tr><th>Initial quality of a body of evidence</th><th>Study design</th><th>Lower if</th><th>Higher if</th></tr>
<tr><td>High</td><td>RCT</td><td rowspan="4">Risk of Bias
-1 Serious
-2 Very serious
Inconsistency
-1 Serious
-2 Very serious
Indirectness
-1 Serious
-2 Very serious
Imprecision
-1 Serious
-2 Very serious
Publication bias
-1 Likely
-2 Very likely</td><td rowspan="4">Large effect:
+1-Large **
+2-Very large ***
+1-Evidence of a Dose response gradient
All plausible residual confounding
+1 Would reduce a demonstrated effect
+1 Would suggest a spurious effect if no effect was observed</td></tr>
<tr><td>Moderate</td><td>Quasi-RCT</td></tr>
<tr><td>Low</td><td>Observational study</td></tr>
<tr><td>Very low</td><td>Any other evidence</td></tr>
</table>

The highest possible score is High (4) and the lowest possible score is Very low (1). Thus, for example, randomised trials with a strong association would not move up a grade.

** A relative risk of >2 (< 0.5), based on consistent evidence from two or more observational studies, with no plausible confounders

*** A relative risk of > 5 (< 0.2) based on direct evidence with no major threats to validity

Table S4: Basic Characteristics of Trials Included

Author, Year	Study Design	Target Population	Trial was Registered?	Randomization Method	Not experienced acupuncture pretreatment?	Blinded?	Blinding Survey?	Sample Size Calculation? /Power (%)	Sample size/dropout rate (%)	Multicenter trial? Acupuncturist versus participants
Liang, 2011[25]	Parallel	Volunteer	Yes	Computer	Unclear	SB(PB)	Yes	Yes/90%	190/6.32	No, NM
Sahin, 2010[26]	Parallel	Outpatient	No	NM	Yes	DB(PAB)	No	No	31/6.45	No, NM
Itoh, 2007[27]	Parallel	Outpatient	No	Computer	Unclear	DB(PAB)	Yes	No	40/22.5	No, NM
Zhu, 2002[28]	Crossover	Volunteer	No	NM	Yes	SB(PB)	No	No	29/0	No, NM
Fu, 2009[29]	Parallel	Outpatient	Yes	Computer	Unclear	SB(PB)	No	No	117/4.27	No, NM
Tough, 2010[30]	Parallel	Outpatient	No	Computer	Yes (79% of subjects)	DB(PAB)	No	Yes/80% underpower	41/17	No,
Molsberger, 2010[31]	Parallel	Outpatient	Yes	Central	Yes	DB(PAB)	No	Yes/90%	289/30.3	Yes, 31:289
Lathia, 2009[32]	Parallel	Outpatient	No	Table	Yes	DB(PAB)	No	Yes/80% 36 per arm needed	31/9.7	No, 2:31
Dyson, 2007[33]	Parallel	Wheelchair-user	No	NM	Yes	DB(PAB)	No	No	17/0	No, 2:17
Guerra, 2004[34]	Parallel	Outpatient	No	Computer	Yes	DB(PAB)	No	Yes/80%	130/7.7	No, 2:130
Kleinhenz, 1999[35]	Parallel	Sportsmen	No	Central	Unclear	DB(PAB)	No	Yes/80%	52/16.6	No, 2:52
He, 2004[36]	Parallel	Office	No	Drawing	Unclear	DB(PAB)	No	No	24/0	No, 1:24
Nabeta, 2002[37]	Parallel	Volunteer	No	Computer	No	SB(PB)	Yes	No	34/0	No, 5:34
Goldman, 2008[38]	Parallel	Repetitive hand users	Yes	Computer	Yes	DB(PAB)	Yes	Yes/80%	123/13.3	No, 8:123
Fink, 2002[39]	Parallel	Volunteer	No	Table	Yes	DB(PAB)	No	Yes/80% enough	45/2.99	No, 1:45
Molsberger, 1994[40]	Parallel	Volunteer	No	Unclear	Yes	SB(PB)	No	Yes/90% 72 subjects needed	48/0	No, 1:48
Hasegawa, 2013[41]	Parallel	Outpatient	Yes	Computer	Yes	DB(PAB)	Yes	Yes/80%	80/0	No, 1:80
Vas, 2012[42]	Parallel	Outpatient	Yes	Computer	Yes	DB(PAB)	No	Yes	205/23.6	Yes, >1:205
Kennedy, 2008[45]	Parallel	Waitlist	No	Computer	Unclear	DB(PAB)	Yes	No	48/17	No, 3:48

Miyazaki, 2009[43]	Parallel	Outpatient	No	NM	Yes	TB(PAB)	Yes	underpower	14/6.67	No, 1:14
Cherkin, 2009[44]	Parallel	Outpatient	Yes	Computer	Yes	DB(PAB)	Yes	Yes/90%	477/3.98	No, 6:477
Haake, 2007[46]	Parallel	Volunteer	Yes	Computer	Yes	DB(PAB)	Yes	Yes	774/3.75	Yes,>340:774
Itoh, 2006[47]	Crossover	Outpatient	No	Computer	Unclear	DB(PAB)	Yes	No	26/7.7	No, 1:26
Inoue, 2006[48]	Parallel	Outpatient	No	Computer	No	DB(PAB)	Yes	No	31/0	No, 1:31
Brinkhaus, 2006[49]	Parallel	Volunteer	Yes	Computer	Yes	DB(PAB)	Yes	Yes/90%	222/2.25	Yes, >30:222
Molsberger, 2002[50]	Parallel	Inpatient	No	Computer	Yes	DB(PAB)	No	Yes/90%, 380 needed	126/0	No, 1:126
Leibing, 2002[51]	Parallel	Outpatient	No	Computer	Unclear	DB(PAB)	No	Yes/90%, 140 needed	85/28.2	No, 1:85
Mendelson, 1983[52]	Crossover	Volunteer	No	NM	Unclear	DB(PAB)	No	Yes	95/18.95	No, 1:77
Horng, 2013[53]	Parallel	Outpatient	Yes	Computer	Unclear	SB(AB)	No	No	28/21.42	No, 1:22
Mavrommatis, 2012[54]	Parallel	Outpatient	No	Computer	Yes	DB(PAB)	No	Yes/90%	80/1.25	No, 1:80
Lu, 2010[56]	Parallel	NM	No	NM	Unclear	SB(PB)	No	No	20/0	No, 1:20
Suarez, 2010[55]	Parallel	NM	No	Computer	Yes	DB(PAB)	Yes	Yes/99%	455/0	No, 6:455
Jubb, 2008[58]	Parallel	Outpatient	No	Computer	Yes	DB(PAB)	Yes	Yes/80%	68/8.82	No, 1:68
Itoh, 2008[59]	Parallel	Outpatient	No	NM	Unclear	DB(PAB)	Yes	No	30/20	No, 2:30
Foster, 2007[60]	Parallel	Outpatient	Yes	NM	Unclear	DB(PAB)	No	Yes/80%	236/3.39	Yes, 37:236
Scharf, 2006[61]	Parallel	Outpatient	Yes	Computer	NM	DB(PAB)	Yes	Yes/90%	697/0.09	Yes, 320:697
Witt, 2005[62]	Parallel	Volunteer	No	Central	Unclear	DB(PAB)	Yes	Yes/80%	226/0.09	Yes, >28:224
Vas, 2004[63]	Parallel	Outpatient	No	Computer	Yes	DB(PAB)	No	Yes/80%	97/9.28	No, 1:97
Berman, 2004[64]	Parallel	Volunteer	No	Computer	Yes	DB(PAB)	No	Yes	381/25.72	Yes, 7:283
Takeda, 1994[65]					Unclear	DB(PAB)			40/0	
Ashin, 2009[57]	Parallel	Outpatient	No	NM	NM	SB(PB)	No	Yes/95%	40/7.5	No, 1:40
Fink, 2001[66]	Parallel	Volunteer	No	Computer	Unclear	DB(PAB)	No	No	45/6.67	No, 1:67
Gaw, 1975[67]	Parallel	Outpatient	No	NM	NM	DB(PAB)	No	No	40/2.5	No, 1:40
Zanette, 2008[68]	Parallel	Outpatient	No	Computer	Yes	DB(PAB)	No	No	40/25	No, 1:40
Tam, 2007[69]	Parallel	Outpatient	Yes	Computer	Yes	DB(PAB)	No	No	36/19.4	No, 1:36
Harris, 2009[70]	Parallel	Outpatient	No	Computer	Yes	DB(PAB)	Yes	No	20/0	No, 1:20
Harris, 2008[71]	Parallel	Outpatient	No	Computer	Yes	DB(PAB)	No	No	10/0	No, 1:10
Harris, 2005[73]	Parallel	Volunteer	No	Computer	Yes	DB(PAB)	Yes	Yes/80% underpower	114/33.3	No, 1:114
Martin, 2006[72]	Parallel	Outpatient	No	NM	Yes	DB(PAB)	Yes	Yes/80%	50/2	No, 2:50
Assefi, 2005[74]	Parallel	Volunteer	Yes	Computer	Yes	DB(PAB)	Yes	Yes/80%	100/14	No, 8:100

								enough		
Tekin, 2013[75]	Parallel	NM	No	Computer	NM	DB(PAB)	No	Yes/80% enough	46/15.2	No, 1:46
Couto, 2013[76]	Parallel	Outpatient	Yes	NM	Yes	DB(PAB)	No	Yes/90% enough	56/3.84	No, 1:78
Chou, 2011[77]	Parallel	Outpatient	No	NM	Yes	DB(PAB)	No	No	45/0	No, 1:45
Tsai, 2010[78]	Parallel	Outpatient	No	Computer	Yes	DB(PAB)	No	No	35/0	No, 1:35
Sun, 2010[79]	Parallel	Outpatient	No	Computer	NM	DB(PAB)	No	Yes/90% enough	35/2.9	No, 1:35
Shen, 2009[80]	Parallel	Outpatient	No	Computer	Yes	DB(PAB)	Yes	Yes/86%	28/0	No, 1:28
Chou, 2009[81]	Parallel	Outpatient	No	Computer	Yes	DB(PAB)	No	No	20/0	No, 1:20
Shen, 2007[82]	Parallel	Outpatient	No	NM	NM	DB(PAB	Yes	underpower	15/0	No, 1:15
Goddard, 2002[83]	Parallel	Volunteer	No	Table	NM	DB(PAB)	No	No	18/0	No, 1:18
Birch, 1998[84]	Parallel	Outpatient	No	NM	Yes (83% of subjects)	DB(PAB)	No	No	46/21.7	No, 1:46
McMillan, 1997[85]	Parallel	Outpatient	No	NM	NM	DB(PAB)	No	Yes/90%	20/0	No, 1:30
Diracoglu, 2012[87]	Parallel	Outpatient	No	Computer	NM	DB(PAB)	No	No	52/3.85	No, 1:50
Smith, 2007[86]	Parallel	Outpatient	No	Computer	NM	DB(PAB)	No	No	27/3.7	No, 1:27

AB: Assessor blinded; Central: Randomized by central telephone randomization procedure; Computer: Randomized by computer software; Table, Randomized by a table of random numbers; NM: not mentioned; PAB: Patient and assessor blinded; PB: Patient blinded; SB, single blind.

Table S5: Demographics and Outcome Assessment

Study, country	Types of condition	Female/Male (Female%)	Age, range (mean±SD) (year)	Pain at baseline, range (mean±SD) on VAS 10 cm	Duration of Disease, range (mean±SD) months	Outcome measure/follow-up
Liang, 2011[25] China	NP (Chr, NS)	129/49 (72.5%)	18-60, (36.98±9.89)	3-7, (5.40±1.74)	>6, (47.62±43.68)	(1) Pain: VAS 10 cm<br>(2) Function: NPQ (China)/im, 3 mths
Sahin, 2010[26] Turkey	NP (Chr, NS)	26/3 (89.3%)	18-65, (36.68±9.92)	>3, (6.72±1.71)	>3	(1) Pain: VAS 10 cm/im, 3 mths
Itoh, 2007[27] Japan	NP (Chr)	29/11 (72.5%)	47-80 (63.61±10.62)	6.8±1.7	>6, (35.4±35.88)	(1) Pain: VAS 10 cm<br>(2) Function: NDI 50/im, 3 wks
Zhu, 2002[28] Australia	NP (Chr)	14/15 (48.3%)	31-70, (49.43±10.35)	4.59±2.18,	>6, (69.40±86.48)	(1) Pain: VAS 10 cm<br>(2) Function: NDI 40/1 wk
Fu, 2009[29] China	NP (Chr, CS)	85/32 (72.6%)	18-60, (34.89±10.31)	5.36±1.75	>6, (42.41±40.34)	(1) Pain: VAS 10 cm<br>(2) Function: NPQ 100/im, 1 mth, 3 mths
Tough, 2010[30] UK	NP (Acute, Whiplash)	24/17 (58.5%)	18-63, (35.58±10.93)	>=3, (4.95±1.60)	0.75-4, (1.75±1.12)	(1) Pain: VAS 10<br>(2) Function: NDI/im
Molsberger, 2010[31] Germany	SP (Chr)	264/156 (62.3%)	25-65, (50.8±9.7)	(6.62±1.38)	1.5, -2 yrs (10.6±9.5)	(1) Pain: VAS<br>(2) Shoulder mobility/im, 3 mths
Lathia, 2009[32] USA	SP (Chr)	28/3 (97%)	>=18, (61.9±4.3)	NM	>=8 wks (43.1±17)	(1) Pain: SPADI-pain score<br>(2) Function: SPADI-disability/im
Dyson, 2007[33] USA	SP (Chr, after spine core injury)	2/15 (11.7%)	18-70, (38.7±11.1)	(4.9±2.3)	>=3, (135.6±108)	(1) Pain: NRS-pain 10<br>(2) WUSPI/im, 5 wks
Guerra, 2004[34] Spain	SP (Chr, tendinitis/capsulitis)	97/130 (74.62%)	>=18, (59.15±11)	(6.2±2.2)	>=3, (6.25±7.48)	(3) Pain: VAS<br>(4) Function: SPADI-disability/im, 2.7 mths, 5.7 mths
Kleinhenz, 1999[35] Germany	SP (rotator cuff tendinitis)	21/31 (40.4%)	18-50, (35.62±9.28)	NM	>=1	(1) Pain: Constrant Murley score- pain scale 15 points/im, 3 mths
He, 2004[36] Norway	NP & SP (Chr)	24/0 (100%)	20-50, (47±9)	5.33±0.91	>3, (144±108)	(1) Pain: VAS 100 mm/im, 6 mths, 3 yrs
Nabeta, 2002[37] Japan	NP & SP (Chr)	24/10 (70.6%)	20-63, (32.5±11.54)	5.24±2.41	NM	(1) Pain: VAS 100 mm/9 days
Goldman, 2008[38] UK	AP (65% tendonitis/epicondylitis) Chr	61/62 (49.6%)	>=18, (36.17±10.58)	>=3, (4.85±1.95)	>=3, (60% of participants >=12)	(1) Pain: NRS 10 points<br>(2) Function: Upper Extremity Function Scale/im, 1 mth

Fink, 2002[39] Germany	AP (Chr epicondylitis)	49/31 (61.3%	(52.07±9.35)	(17.89±1.35) on VRS 25	= (median 9)	(1) Pain: VRS 25<br>(2) Function: DASH/im, 2 mths
Molsberger, 1994[40] Germany	AP (Chr epicondylitis)	26/48 (54.2%)	47.8	6.4	2 mths-12 yrs (15.4±1.24)	(1) Pain: VAS 10/im
Hasegawa, 2013[41] Brazil	LBP (Acute, NS)	51/29 (63.8%)	18-65, (45.45±10.48)	4-8, (6.61±1.42)	<1, (15.25±11.35 days)	(1) VAS 10 cm<br>(2) Function: RMQ/0 day
Vas, 2012[42] Spain	LBP (Acute, NS)	161/114 (58.5%)	18-65, (42.67±11.11)	(7.04±1.78)	<0.5, (6.06±3.72 days)	(1) Pain: VAS 100 mm<br>(2) Function: RMQ/1 wks, 10 wks, 46 wks
Kennedy, 2008[45] UK	LBP (Acute, NS)	25/23 (52.1%)	18-70, (45.55±11.14)	(5.94±0.59)	<3	(1) Pain: VAS 100 mm<br>(2) Function: RMQ/im, 3 mths
Miyazaki, 2009[43] Japan	LBP (Chr, NS)	0/14 (0%)	>=20, (20.86±0.56)	(3.55±2.1)	>=3	(1) Pain: VAS<br>(2) Function: RDQ 24/im
Cherkin, 2009[44] USA	LBP (Chr, NS)	(62%)	18-70, (47±13)	NM	>=3	(1) Function: RMQ/1 wks, 18 wks, 44 wks
Haake, 2007[46] Germany	LBP (Chr, NS)	585/577 (50.3%)	18-86, (50±15)	(67.77±13.91) on CPGS 100	>=6	(1) Pain: CPGS 100<br>(2) Function: HFAQ (lower better) 100/1 wks, 7 wks, 20 wks
Itoh, 2006[47] Japan	LBP (Chr, S),	17/9 (65.4%)	65-91, (76.01±8.37)	(6.69±1.30)	>=6, (57.24±60)	(1) Pain: VAS 10 cm<br>(2) Function: RMQ/im, 0.5 mth
Inoue, 2006[48] Japan	LBP (Chr, S) lumbar vertebral arthritis	10/21 (32.6%)	NM, (69.03±7.17)	(6.1±1.1)	NM (83.51±42.76)	(1) Pain: VAS 10 cm/im
Brinkhaus, 2006[49] Germany	LBP (Chr, NS)	202/96 (67.8%)	40-75, (59±9)	>=4, (6.48±1.4)	>=6, (176.4±133.2)	(1) Pain: VAS 100 mm<br>(2) Function: PDI/im, 18 wks, 44 wks
Molsberger, 2002[50] Germany	LBP (Chr, NS)	89/97 (47.8%)	20-60, (50±7)	>=5, (6.6±1.5)	>=6, (118.8±93.6)	(1) Pain: VAS 100 mm/im, 3 mths
Leibing, 2002[51] Germany	LBP (Chr, NS)	76/55 (58%)	18-65, (48.1±9.7)	(5.2±1.9)	>=6, (115.2±98.4)	(1) Pain: VAS 100 mm<br>(2) Function: PDI/im, 9 mths
Mendelson, 1983[52] Australia	LBP (Chr, S) arthritis	40/37 (51.2%)	(54.02±11.86)	(5.2±4)	(146.3±130.2)	(1) Pain: VAS 100 mm/im
Horng, 2013[53] China	KOA	21/7 (75%)	62-83, (67.18±3.6)	>=3, (4.68±1.1)	NM	(1) Pain: VAS 100 mm<br>(2) Function: WOMAC total/im, 1.5 mths
Mavrommatis,	KOA (Chr)	91/29 (75.8%)	(61.79±10.62)	(6.27±1.03)	>=3	(1) Pain: VAS

2012[54] Greece						(2) Function: WOMAC index Version VA3.1/im, 1 mth
Lu, 2010[56] China	KOA	NM	(65.25±8.89)	(5.25±1.17)	NM	(1) Pain: VAS<br>(2) Gait pattern/im
Suarez, 2010[55] USA	KOA (Chr)	338/189 (64.1%)	>=50, (64.45±9.20)	(5.73±2.29)	(109.9±124.8)	(1) Pain: VAS<br>(2) Function: WOMAC function/im, 1.5 mths
Jubb, 2008[58] UK	KOA (Chr)	55/13 (80.9%)	>=18, (65.1±2.02)	(6.15±2.23)	>=6, (117.4±16.51)	(1) Pain: VAS<br>(2) Function: WOMAC function scale/im, 1 mth
Itoh, 2008[59] Japan	KOA (Chr)	27/3 (90%)	61-82, (72.6±7.84)	(6.56±1.09)	>=6, (77.76±73.2)	(1) Pain: VAS<br>(2) Function: WOMAC total/im, 5 wks, 15 wks
Foster, 2007[60] UK	KOA (Chr)	NM	>=50	NM	NM	(1) Pain: WOMAC pain scale<br>(2) Function: WOMAC function/im, 21 wks, 49 wks
Scharf, 2006[61] Germany	KOA (Chr)	475/216 (68.74%)	>= 40, (62.9±10)	NM	>=6, (63.70±69.75)	(1) Function: WOMAC total/im, 11 wks
Witt, 2005[62] Germany	KOA (Chr)	195/99 (66%)	50-70, (64±6.5)	>=4, (6.53±1.45)	(110.4±94.8)	(1) Pain: WOMAC pain scale<br>(2) Function: WOMAC function/im, 4.5 mths, 10.5 mth
Vas, 2004[63] Spain	KOA (Chr)	81/16 (83.5%)	45-91, (67.1±10.2)	(5.96±1.25)	>=3, (90.12±103.2)	(1) Pain: VAS<br>(2) Function: WOMAC index/im
Berman, 2004[64] USA	KOA (Chr)	365/205 (64%)	>=50, (65.5±8.6)	(8.94±3.50) on WOMAC pain scale 20	>=5 yrs (49.7% of the patients)	(1) Pain: WOMAC pain<br>(2) Function: WOMAC function/im
Takeda, 1994[65] Japan	KOA	20/20 (100%)	NM	NM	NM	(1) Pain: MPQ<br>(2) Function: WOMAC function/im
Ashin, 2009[57] Pakistan	KOA (Chr)	28/12 (70%)	(51.25±8.34)	(8.28±8.7)	(18.98±15.25)	(1) Pain: VAS 100 mm<br>(2) Function: WOMAC total/im
Fink, 2001[66] Germany	HOA (Chr)	43/22 (66.15%)	(62.6±9.1)	(5.49±2.13)	>=6, (61.83±45.73)	(1) Pain: VAS<br>(2) Function: Hip function index/im, 2 mths
Gaw, 1975[67] USA	OA (hip, knee,	NM	42-81	mean 3.25 on total 4 points	NM	(1) Pain: 4 points scale<br>(2) Activity/im

	lumbar, cervical, thoracic, finger joints)			scale		
Zanette, 2008[68] Brazil	RA (Chr)	37/3 (92.5%)	18-75, (49.8±11.7)	(6.5±2.62)	>=6, (7-16 yrs)	(1) Pain: VAS/im, 1 mth
Tam, 2007[69] China	RA (Chr)	29/7 (80.6%)	>=18, (57.4±9.7)	(5.97±2.37)	(109.2±76.44)	(1) Pain: VAS/im
Harris, 2009[70] USA	FM (Chr)	20/0 (100%)	(44.3±13.6)		>=12	(1) Pain: MPQ 45/im
Harris, 2008[71] USA	FM (Chr)	10/0 (100%)	(48±15)	(12.3±4.35) on MPQ	>=12	(1) Pain: MPQ 45/im
Harris, 2005[73] USA	FM (Chr)	106/8 (93%)	(47.4±10.8)	(5.54±2.3)	>=12, (65±51)	(1) Pain: NRS 100/im
Martin, 2006[72] USA	FM (Chr)	49/1 (98%)	(49.8±12.87)	(41.67±9.2) on MPI	NM	(1) Pain: MPI 100<br>(2) Function: FIQ 0-10/im, 1 and 7 mths
Assefi, 2005[74] USA	FM (Chr)	94/2 (98%)	>=18, (47.2±11.64)	>=4, (7±2)	(74.9±56.28)	(1) Pain: VAS 10 cm/im, 3 and 6 mth
Tekin, 2013[75] Turkey	MP (Chr, Upper trapezius muscle)*	31/8 (79.5%)	24-65, (42.5±11.4)	(6.51±1.44)	>=6, (61.1±49.7)	(1) Pain: VAS 10 cm/im
Couto, 2013[76] Brazil	MP (Chr, Upper half body)	78/0 (100%)	19-50, (34.6±5)	(6.62±1.09)	>=3	(1) Pain: VAS 10 cm/im
Chou, 2011[77] China	MP (Chr, Upper trapezius muscle)	23/22 (51.1%)	22-58, (34.1±9)	>=5, (7.53±1.12)	>=3, (6.13±2.23)	(1) Pain: NRS 10/im
Tsai, 2010[78] China	MP (Chr, Upper trapezius muscle)	21/14 (60%)	22-68, (43.9±11.4)	>=5, (7.25±1.4)	3-18, (7.14±4.23)	(1) Pain: NRS 10/im
Sun, 2010[79] China	MP (Chr, Neck)	25/10 (71.4%)	31-66 (Mean 46.7)	4.5-6.5, (Mean 5)	>=1	(1) Pain: MPQ/im, 1 mth, 3 mths
Shen, 2009[80] USA	MP (Chr, jaw muscle)	28/0 (100%)	>=18, (40.3±13.5)	>=4, (5.14±2.48)	>=3	(1) Pain: NRS 10 facial pain/im
Chou, 2009[81] China	MP (Chr, Upper trapezius muscle)	12/8 (60%)	22-53, (35.5±9.92)	(7.4±0.8)	(5.85±3.06)	(1) Pain: NRS 10/im
Shen, 2007[82] USA	MP (Chr, jaw muscle)	14/1 (93.33%)	>=18, (43.1±13.6)	(5.93±2.22)	>=3	(1) Pain: NRS 10 facial pain/im
Goddard, 2002[83] Japan	MP (Chr, jaw muscle)	15/3 (83.33%)	22-52, (35.1±9.13)	(6.39±1.70)	>=3	(1) Pain: VAS/im
Birch, 1998[84]	MP (Chr, Neck)	38/8 (82.6%)	18-65, (mean	(mean 4.8)	>=6, (mean 88.48)	(1) Pain: MPQ/im

Netherland			39.5)			
McMillan, 1997[85] UK	MP (Chr, jaw muscle)	30/0 (100%)	23-53	(3.67±2.26)	>=3	(1) Pain: VAS/im
Diracoglu, 2012 [87] Turkey	MP (Chr, TEM)	43/7 (86%)	18-57, (34.4±11.3)	(6±1.49)	>=1.5	(1) Pain: VAS/im
Smith, 2007[86] UK	MP (Chr, TEM)	24/3 (88.9%)	(40.5±13.6)	mean 4.08	>=6, (51.6±28.8)	(1) Pain: VAS<br>(2) Mouth opening (mm)/im

AP, Arm Pain; Chr, Chronic; COA, Cervical Osteoarthritis; CPGS, Von Korff Chronic Pain Grade Scale; CS, Cervical Spondylosis; DAS, Disease Assessment Score; DASH, Disability of Arm, Shoulder, Hand; FIQ, Fibromyalgia Impact Questionnaire; FM, Fibromyalgia; HAQ, Health Assessment Questionnaire; HFAQ, Hanover Functional Ability Questionnaire; HOA, Hip osteoarthritis; Im, immediately; KOA, Knee osteoarthritis; LBP, Low back pain; MP, Myofascial pain; MPI, Multidimensional Pain Inventory; MPQ, McGill Pain Questionnaire; Mth, month; NM, not mentioned; NS, non-specific; NDI, Neck Disability Index; NP, Neck pain; NPQ, Northwick Neck Pain Questionnaire; ODI, Oswestry Disability Index; PDI, Pain Disability Index; PPT, Pressure Pain Threshold; QoL, Quality of Life; RA, Rheumatoid arthritis; RMQ, Roland Morris Disability Questionnaire; ROM, Range of Motion; SF-36, Short-form 36 health survey; SP, Shoulder Pain; SPADI, Shoulder Pain and Disability Index; tx, treatment; TEM, Temporomandibular; UK, United Kingdom; USA, the United States of America; VAS, Visual analogue scale; VRS, Verbal Rating Scale; Wk, week; WOMAC, Western Ontario and McMaster Universities Osteoarthritis Index; WUSPI, Wheelchair User's Shoulder Pain Index.

* Upper trapezius muscle, a muscle located at the area of shoulder.

Table S6: Detailed STRICTA Information (a)

Study	Rationale of acupuncture	Style of acupuncture (L versus D points, APs, TrPs)/types of stimulation	U versus B/number of needles used/needles used/needle depth	Needle retention time/treatment regimen (described in number of treatment/period) /De Qi*
Liang, 2011[25]	TCM	L/M	B/6/needles (0.3 mm×40 mm)/20 mm	20 min/9 x/3 wks (3 x/wk)/De Qi
	[Sham]	L/No M	B/6/needles (0.18 mm×40 mm)/3 mm	The same/No De Qi
Sahin, 2010[26]	CTs	L and D based on the affected meridians/M and E, low frequency (I -4 Hz), pulse width of 200 µm, interrupted currents with high intensity	B/13/needles (0.25 mm×25 mm)/20 mm	30 min/10 x/4 wks (3 x/wk for 3 wks followed by 1x/wk for 1wk)/De Qi
	[Sham]	L and D/No M and E	The same	The same/No De Qi
Itoh, 2007[27]	TCM, CTs	L and D/M	B/9/needles (0.2 mm ×40 mm)/20 mm	30 min/6 x/6 wks (1 x/wk for 3 wks followed by 3 wks' interval and 1 x/wk for 3 wks)/De Qi
	TrP	TrPs/M	B/2.3/needles (0.2 mm× 50 mm)/20 mm	The same/local twitch response
	Non-TrP	Non-TrP/M	B/2.4/needles (0.2 mm× 50 mm)/20 mm	The same/local twitch response
	TrP *[Sham]*	TPs/M	B/2.6/blunt needles (0.2 mm×50 mm)/0 mm	The same/no response
Zhu, 2002[28]	TCM	L and D/M and E, A strong electrical stimulation was applied on two distal acupoints at a frequency 15–20 Hz and reached patients' tolerance	U and B/4/needles (0.22 mm×40 mm) /correct depth	20 min/9 x/3 wks (3 x/wk)/De Qi
	[Sham]	L and D/M and E, weak electrical stimulation was applied on two distal acupoints at a frequency once per min	U and B/4 needles (0.22 mm×13 mm)/superficial	The same/No De Qi
He, 2004[36]	CTs	L, APs/M and E,	U and B/7-22/needles (0.25-0.35 mm×25-40 mm)/10-30 mm	45 min/10 x/3-4 wks(3 x/wk)/De Qi
	[Sham]	The same/No	The same	The same
Nabeta, 2002[37]	TCM, TrP	L/M	B/2-12 (6.5±2.3)/needles (0.2	>5 min/3 x/3 wks(1x/wk)/De Qi

			mm×40 mm)/20 mm	
	[Sham]	The same/No	The same/blunt needles/0 mm	The same/No
Fu, 2009[29]	TCM	L/M	B/5/needles (0.3 mm×40 mm)/20 mm	20 min/9 x/18 days(1x/2 days)/De Qi
	[Sham]	L/No M	The same/needles (0.22 mm×40 mm)/superficial	The same/No
Hasegawa, 2013[41]	Japanese acupuncture, CTs	L and D/M	B/12/needles (0.20 mm×13 mm)/3-5 mm (angle 15 °)	30 min/5 x/4 wks(2x/wk for first wk followed 1x/wk for 3 wks)/NM
	[Sham]	The same/No	The same	The same
Vas, 2012[42]	TCM	NM/M	B/NM/NM/NM	20 min/5 x/2 wks/NM
		NM/M	B/NM/NM/NM	The same
	[Sham]	NM/M	B/NM/(semiblunted needles) /0 mm	The same
Kennedy, 2008[45]	Western theory, CTs, TI, experts opinion	L and D/M	B/8-13/needles (0.25 mm×40 mm)/5-30 mm	30 min/3-12 x/4-6 wks(1-2x/wk)/De Qi
	[Sham]	The same/No	The same/needles (0.3 mm×40 mm)/0 mm	The same/No
Miyazaki, 2009[43]	Japanese	L/No	U/1/needles (0.2 mm×0.6 mm) /0.3 mm Superficial	NM/1x/NM
	[Sham]	L/No	The same/no needles/0 mm non-penetration	The same
Cherkin, 2009[44]	TCM	L and D/M	B/10.8/needles (0.25 mm×40 mm)/10-30 mm	18 min/10 x/7 wks(2 x/wk for 3 wks followed 1 x/wk for 4 wks)/De Qi
	TCM, IT	The same	B/8/the same	The same
	CTs*[Sham]*	The same	B/NM/guidetube containing a toothpick/0 mm	The same/No
Haake, 2007[46]	TCM, IT, CTs	L/M	B/14-20/needles (0.25 mm×40 mm or 0.35 mm×50 mm) /5-40 mm	30 min/10 x/5 wks(2 x/wk for 5 wks followed 5 additional x if)/De Qi
	Literatures, experts*[Sham]*	L/No M	The same /1-3 mm Superficial	The same/No
Itoh, 2006[47]	TrP	L, TrPs/M	U or B/3.6/needles (0.2 mm×50 mm)/10-40 mm	10 min/1 x/wk for 3 wks/local twitch response
	TrP*[Sham]*	The same/No	U or B/3.5/blunt needles (0.2	The same/No

			mm×50 mm)/0 mm	
Inoue, 2006[48]	Most painful point	L/M	U/1/needles (0.18 mm×40 mm)/20 mm	NM/1 x/De Qi
	The same*[Sham]*	The same/No	U/1/guide tube without needle/0 mm	The same/No
Brinkhaus, 2006[49]	TCM, experts	L and D, APs, TrPs/M	B/>=12/needles (0.18 mm×40 mm)/20 mm	30 min/12 x/8 wks (2x/wk for first 4 wks followed by 1x/wk in 4 wks)/De Qi
	The same*[Sham]*	L/No M	B/12/needles (0.18 mm×20-40 mm)/0 mm	The same/No
Molsberger, 2002[50]	TCM, literature	L and D, TrPs/M	B/16/NM/10-100 mm	30 min/12 x/4 wks (3x/wk)/De Qi
	[Sham]	L/NM	B/16/NM/<10 mm, superficial	The same/No
Guerra, 2004[34]	TCM, experts	L, D/E	U/4/needles (0.25 mm× 50 mm)/33 mm	15 min/8x/8 wks (1x/wk)/De Qi & light muscular twitching
	[Sham]	The same/No E	The same/retractable needles/0 mm non-insertion	The same/No
Leibing, 2002[51]	TCM, guideline	L, APs/M	B/27/needles (0.30 mm×40 mm)/10-30 mm, ear needles were ring-shaped	30 min/20 x/12 wks (5x/wk for first 2 wks followed by 1x/wk for 10 wks) /De Qi
	Literature *[Sham]*	L/No M	The same/Superficial	The same/No
Mendelson, 1983[52]	TCM, literature	L/M	B/10-18/NM/NM	30 min/8 x/4 wks (2x/wk)/De Qi
	[Sham]	L/No M	The same/Superficial	The same/No
Horng, 2013[53]	TI, Literature	L and D/E (100 Hz for reduction mode and 40 Hz for enhancement mode)	U/>3/electrode	6 min/6 x/3 wks (2x/wk)/muscle contraction
	[Sham]	The same/No E	The same	The same/No any feeling
Mavrommatis, 2012[54]	TCM, TI	L and D/M and E (M for the first 2x and E for last 2x) (2-6 Hz)	B/18/needles (0.30 mm×30 mm)/NM	30 min/4 x/8 wks (biweekly)/De Qi
	[Sham]	The same/No	The same/0 mm	The same/No
Lu, 2010[56]	TCM	L and D/E (0.5 mA, 2 Hz, 1 ms)	B/10/NM/10-15 mm	30 min/1 x/De Qi
		The same/No E	The same	The same/No
Suarez, 2010[55]	TCM	L and D/E (dense 50 Hz, disperse 15 Hz, 20 cycles/minute, Voltage 5-60 V)	B or U/7-14/needles (0.30 mm×30-50 mm) and 15 mm for AP/5-40 mm	NM
	[Sham]	L and D/E (very weak)	B or U/7-14/needles were thinner and shorter/superficial	NM
Jubb, 2008[58]	TCM, CTs	L and D/M and E (2-10 Hz)	U/9/needles (0.30 mm×30 mm)	30 min/10 x/5 wks (2x/wk)/De Qi

			/10-15 mm	
	CTs*[Sham]*	L and D/No M or E	U/9/retractable needles/0 mm	The same/No
Itoh, 2008[59]	TCM, CTs	L/M	U/6/needles (0.2 mm×40 mm) /10 mm	30 min/5x/5 wks (1x/wk)/De Qi
	TrP	L/M	U/3.3/needles (0.2 mm×50 mm) /10-30 mm	The same/local switch response
	TrP*[Sham]*	L/sham M	U/3.1/blunt needles /0 mm non-penetration	The same/No sensation
Foster, 2007[60]	TCM	NM/M	NM	/6 x/3 wks (2x/wk)/De Qi
	[Sham]	The same/No	The same/retractable needles/0 mm non-penetration	The same/No
Witt, 2005[62]	TCM, TrPs, experts, societies	L and D, TrPs, APs/M	U and B/8-16/needles/NM	30 min/12x/8 wks (2x/wk for the first 4 wks followed by 1x/wk for the remaining 4 wks)/De Qi
	[Sham]	D/No M	NM/8/needles/superficial	The same/No
Vas, 2004[63]	TCM, TI	L and D/E	U and B/8-15/needles (0.30 mm ×45 mm)/NM	NM/12 x/12 wks (1x/wk)/De Qi
	[Sham]	The same/E	The same/retractable needles /0 mm	The same/No
Berman, 2004[64]	TCM, CTs	L and D/E (at knee points Xiyan, 8 Hz, 0.5 ms pulse width, 20 min)	U and B/9 or 18/needles (0.25 mm×38 or 25 mm)/7.6 to 25.4 mm	20 min/23 x/26 wks (2x/wk for 8 wks followed by 1x/wk for 2 wks, 1x/2 wks for 4 wks, and 1x/4 wks for 12 wks)/De Qi
	CTs*[Sham]*	The same/No E	The same/mock plastic needle guiding tube/0 mm non-penetration	The same/No
Takeda, 1994[65]			U/5/	/3 x/3 wks (1x/wk)/De Qi
	[Sham]		/superficial	
Gaw, 1975[67]	TCM, TI, experts	NM/M	NM/NM/needles NM/NM	30 min/8x/3 wks (3x/wk)/De Qi
	[Sham]	NM/No M	The same/insertion same depth	The same/No
Fink, 2001[66]	Literature	L and D/M	U/12/needles (0.3 mm×60 mm) /NM	20 min/10 x/3 wks/De Qi
	[Sham]	The same/No M	The same	The same
Scharf, 2006[61]	TCM	L, D, TrP/M	U/7-15/needles (diameter 0.3 mm)/regular depth	20-30 min/10-15x/6 wks (1-2x/wk)/De Qi
	[Sham]	L, D/No M	U/10/the same/5 mm superficially	The same/No

Ashin, 2009[57]	TCM	L, D/E	U/6/needles (30 mm)/10-30 mm	20-25 min/10x/10 d (1x/d)/De Qi
	[Sham]	The same/No E	The same/5-10 mm	The same/No
Zanette, 2008[68]	TI	L and D/M	U and B/16-30/needles (0.25 mm×40 mm) /NM	40 min/10 x/5 wks (2x/wks)/De Qi
	Literature*[Sham]*	L and D/No M	U and B/fewer/needles (0.25 mm×15 mm) /<=2 mm superficial	20 min/The same/No
Tam, 2007[69]	TCM	L and D/M and E (dense 4 Hz, disperse 20 Hz)	B/12/needles (0.25 mm×25 or 40 mm) /10 or 20 mm	30 min/20 x/10 wks (2x/wks)/De Qi
	TCM	L and D/M	B/12/needles (0.25 mm×25 or 40 mm) /10 or 20 mm	30 min/20 x/10 wks (2x/wks)/De Qi
	[Sham]	L and D/No	The same/<=2 mm superficial	The same/No
Molsberger, 2010[31]	TCM, TIs, experts	L and D/M	U/5-10/needles (0.3 mm×30 mm)/10-20 mm	20 min/15 x/6 wks (1-3x/wk)/De Qi
	[Sham]	The same/No	U/8/same needles/<=5 mm superficial	The same/No
Lathia, 2009[32]	TCM	L and D/M	NM/8-16/needles (0.2 mm×NM)/3-25 mm	30 min/12 x/6 wks (2x/wk)/De Qi
	Fixed protocols	NM/M	NM/7 /The same	The same/De Qi
	[Sham]	NM/No	NM/7 /retractable needles/0 mm non-penetration	The same/No
Dyson, 2007[33]	TCM, Fixed, TrPs	L and D, TrPs/M	U/9-12/needles NM/10-30 mm	20 min/10 x/5 wks/De Qi
	CTs*[Sham]*	L and D/No	U/8/The same/superficial	The same/No
Kleinhenz, 1999[35]	TCM, CTs	L and D/M	U/12/needles NM /NM	20 min/4 x/4 wks/De Qi
	[Sham]	The same	U/12/retractable needles/0 mm non-penetration	The same/No
Goldman, 2008[38]	TCM, Literature, TrP	L and D, TrPs/M	U or B/8-38/needles (0.25 mm×NM)/standard depth	20 min/8 x/4 wks/De Qi
	[Sham]	The same/No	The same/retractable needles/0 mm non-penetration	The same/No
Fink, 2002[39]	TCM, TI	L and D, TrP/M	U/5/needles (0.25 mm×40 mm) /standard depth	25 min/10 x/5 wks (2x/wk)/De Qi
	[Sham]	The same/M	The same	The same/No
Molsberger, 1994[40]	TCM	D/M	U/1/NM/20 mm	5 min/1 x/De Qi
	[Sham]	On the back/No	The same/0 mm non-insertion	The same/No

Tough, 2010[30]	TrP	TrPs/M	B/NM/needles (0.25 mm×30 or 40 mm) /standard depth	NM/2-6x/6 wks (1x/wk for 6 wks)/sensation
	[Sham]	The same	The same/blunt needles/0 mm non-penetration	The same/No
Harris, 2009[70]	TI	L, APs/M	U/9/NM/20 mm	25 min/9 x/4 wks (2x/wk)/De Qi
	[Sham]		The same/0 mm non-insertion	/No
Harris, 2008[71]	TI	L, APs/M	U/9/needles (0.25 mm× 50 mm)/20 mm	25 min/9 x/4 wks (2-3x/wk)/De Qi
	[Sham]		The same/0 mm non-insertion	/No
Harris, 2005[73]	TI	L, APs/M	U/9/needles (0.25 mm× 38 mm)/20-30 mm	25 min/18x/13 wks (1x/wk for 3 wks followed by 2x/wk for 3 wks and 3x/wk for 3 wks)/De Qi
	[Sham]	The same/No M	The same/20-30 mm	The same/No
	[Sham]	Irrelevant points/M	The same/20-30 mm	The same/No
	[Sham]	Irrelevant points/No M	The same/20-30 mm	The same/No
Martin, 2006[72]	TCM,	L and D/E (2 Hz, 12 Hz at LI4 and ST36)	B/18-20/NM /into muscles	20 min/6x/3 wks (2x/wk)/NM
	[Sham]			
Assefi, 2005[74]	TCM, Experience, discussion	NM /M	NM/7-14/needles (NM× 34-40 mm)/20-30 mm /standard depth	30 min/24 x/12 wks (2x/wk) /NM
	[Sham]	NM /M or No	The same/insertion or non-insertion	The same
Tekin, 2013[75]	TrP	TrPs/NM	B/6/needles (0.25 mm× 25 mm)/move forward trigger point was reached	/6 x/4 wks (2x/wk for first 2 wks followed by 1x/wk for last 2 wks)/muscle twitch response
	[Sham]	TrPs/No	The same/blunt needles/0 mm non-penetration	The same/No response
Couto, 2013[76]	TrP	TrPs/No	U/4.33/needles (0.25 mm× 25 mm)/intramuscular	30 min/8x/4 wks (2x/wk)/muscle twitch response
	TrP*[Sham]*	TrPs/No	U/NM/no needles, but electrodes/0 mm non-insertion	The same/No sensation
Chou, 2011[77]	TCM, CTs, experts	D/M (screw in and out)	U/2/needles (0.3 mm× 25-37 mm)/regular depth	30 min/1x/muscle twitch response and De Qi
	[Sham]	D/No	The same/0 mm non-insertion	The same/No sensation
Tsai, 2010[78]	TrP	L and D, TrPs/M (rapidly insert in and out, with as many times as possible)	U/>=1/a 5 ml syringe with a 25-hypodermic needle (0.5	2 min/1x/muscle twitch response

			mm×35 mm)/intramuscular	
	[Sham]	The same/M	The same/superficial	The same/No sensation
Sun, 2010[79]	TCM	L/M	B/6/needles (0.25 mm× 25-35 mm)/regular depth	20 min/6x/3 wks (2x/wk)/De Qi
	[Sham]	L/No	The same/2 mm superficially	The same/No
Shen, 2009[80]	TCM, CTs	D/M	U/1/needles (0.30 mm× 30 mm)/10-20 mm	20 min/1x/NM
	[Sham]	D/No	U/1/blunt needle/0 mm non-insertion	The same/NM
Chou, 2009[81]	TCM, CTs, experts	D/M (screw in and out)	U/2/needles (0.3 mm× 25-37 mm)/regular depth	30 min/1x/muscle twitch response and De Qi
	[Sham]	D/No	The same/0 mm non-insertion	The same/No sensation
Shen, 2007[82]	TCM, CTs	D/M	U/1/needles (0.30 mm× 30 mm)/10-20 mm	20 min/1x/NM
	[Sham]	D/No	U/1/blunt needle/0 mm non-insertion	The same/NM
Goddard, 2002[83]	TCM, CTs	D/M (twirled once for 5 seconds)	B/4/needles NM/10-30 mm	30 min/1x/De Qi
	[Sham]	The same/M	The same/2-4 mm Superficial	The same/No
Birch, 1998[84]	Japanese, CTs, literatures	L and D/NM	B/19/needles (0.18 mm× 25-37 mm)/2-3 mm **Superficially**	30 min/14x/12 wks (2x/wk for 4 wks followed by 1x/wk for next 4 wks, and 1 x/2 wks for 4 wks)/NM
	literatures*[Sham]*	L and D/NM	B/19/needles (0.18 mm× 25-37 mm)/2-3 mm **Superficially**	The same
McMillan, 1997[85]	TrP	L, TrP /M (rapidly insert in and out)	NM/NM/a syringe with 0.4 mm hypodermic needle/intramuscular	1-2 min/1x/NM
	[Sham]	The same/M	The same/percutaneously	The same
Diracoglu, 2012[87]	TrP	L, TrP /M (rapidly insert in and out) intramuscular stimulation 3-5 times	NM/NM/needles (0.22 mm× 30 mm)/intramuscular	NM/3x/3 wks (1x/wk)/NM
	[Sham]	L/M (rapidly insert in and out) intracutaneous stimulation	The same/intracutaneous	The same
Smith, 2007[86]	TCM, CTs	L, TrP /M	B/2/needles (0.35 mm× 70 mm)/6-12 mm	20 min/6x/3 wks (2x/wk)/pain felt
	[Sham]	L, TrP /M	The same/retractable needles/0 mm non-insertion	The same/No

AP: Auricular point; B: Bilateral; CT: controlled trials; D: Distal; L: Local; E, Electricity; LBP: Low back pain; M: Manual; Mth: month; NM: not mentioned; TI: Textbooks information; TrP: Trigger point; U: Unilateral; Wk: week; TCM: traditional Chinese Medicine; Yr: year.

*De Qi indicates a local sensation of heaviness, numbness, soreness or paresthesia that accompanies the insertion and manipulation of needles during acupuncture.

Table S7: Detailed STRICTA Information (b)

Study	Acupoints used in the trial	Acupuncturist's training	Co-intervention
Liang, 2011[25]	DU14, SI15 and Ex-HN15 (all selected bilaterally) in the cervical region.	NM	infrared irradiation on the cervical region, medication
	[Sham] 1 cm **lateral to** the standard acupuncture points selected in the study group, **superficially**	NM	The same
Sahin, 2010[26]	bilateral Bladder 10 (BL1O), Bladder 60 (BL60), Large intestine 4 (LI4), Triple Energiser 5 (TE5), Gall bladder 20 (GB20), Gall bladder 21 (GB21) and GoverTior 14 (GV14), points used in previous studies	Licensed acupuncturists; > 15 yrs of experience	NM
	[Sham] 1-2 cm **lateral to** the standard acupuncture points selected in the study group	The same	NM
Itoh, 2007[27]	The standard points in the cervical region (local points) were GB 20 and 21, BL 10 and 11, S 12 and 13; standard points on the upper extremity (distal points) were TE 5, LI 4 and SI 3	4 years of acupuncture training and 2 or 7 years of clinical experience.	Medication
	Trigger points, the most important muscles of the cervical and upper extremity	The same	The same
	non-tender point was selected in the same muscle as the trigger point and away from the trigger point by 50 mm.	The same	The same
	[Sham] The methods of choosing trigger **points were the same**, but **no penetration**	The same	The same
Zhu, 2002[28]	GB 20, Go 14, LI 11, LI 10, TB 5 and TB 8 in the neck and arm; GB 21, SI 12 and SI 13 in the shoulder	NM	NM
	[Sham] 2.0 cm **lateral to** the real acupoints in the neck and arm; 3.0 cm lateral to the real points in the shoulder	NM	NM
Fu, 2009[29]	DU14, Ex-HN15, SI15	NM	infrared radiation
	[Sham] The **same points, Superficially** (epidermal layer)	NM	Infrared radiation
Tough, 2010[30]	Trigger points	NM, NM	Plus a program of standardized physiotherapy care, analgesic medication as usual, exercise

	[sham] Trigger points**, non-penetration**	The same	The same
Lathia, 2009[32]	Individualized acupuncture according to TCM, the points used varied between patients and between treatment sessions for each patient. 8-16 points	2 certified rheumatologists, one was trained for 4 years; the other for 1 years, and had over 5 years of experience	Medication (NSAID) as usual, a home exercise program
	Standardized acupuncture according to protocols, 7 points	The same	The same
	[sham] **7 same points** as standardized acupuncture group, **non-penetration**, no manipulation	The same	The same
Dyson, 2007[33]	Standardized: 6 local points (GB21, LI14, 15, SJ14, SI10, 11)and 2 distal points (LI4, 11)<br>Individualized: 1-4 ashi points	2 licensed acupuncturists trained in TCM style acupuncture; >=20 years of experience	Medications as usual
	[sham] **Non-acupuncture points, 2.35 cm lateral to** established meridian, 6 local points and 2 distal points, **Superficially**	The same	The same
Kleinhenz, 1999[35]	Local points: TE14,15, SI9, 11, 12, 14, LI14, 15, BL44, Tianjian, jianquan; Distant points: LI11, SI6, 13, TE3, GB34, ST38; TCM points: L2, H1, PC2	2 experienced acupuncturists	No
	[sham] **The same points,** retractable needles, **non-penetration**	The same	No
He, 2004[36]	Body electroacupuncture ExHN, GB21, BL12, GV14, SI15, SI14; body acupuncture LI14, LI11, GB31; Ear acupressure 5 points	1 experienced (>10 yrs) acupuncturist; NM	Medication
	[Sham] The **same points**, No voltage, 10-40 mm **distal to** real acupoints (body), 4-6 mm (ear), needles	The same	
Nabeta, 2002[37]	Located close to BL-10, GB-20, GB-12, GL-21, and BL-43.	5 well-trained licensed acupuncturists; specially trained	NM
	[Sham] The **same points, no penetration**	The same	NM
Hasegawa, 2013[41]	basic points D, H and I and kidney, bladder and liver points of Yamamoto's method	A member of the Brazilian Medical Association of Acupuncture; 15 years of experience.	Medication (50 mg sodium diclofenac every 8 h)
	[Sham] The **same points, non-penetration**, just handle contact	The same	The same
Vas, 2012[42]	selecting individualized points on the basis of pain characteristics and location	Physicians >700 h of training; 8.5 years of experience	Medications (NSAID, analgesics), posture

			recommendations
	[Sham] **nonspecific** acupuncture **points**	The same	The same
	[Sham] points on the patient's back were selected and momentary pressure applied with a semiblunted needle fitted within a guide tube, **different points, non-penetration**	The same	The same
Kennedy, 2008[45]	GV3, GV4, BL23, BL25, GB29, GB30, GB31, GB34, BL36, BL37, BL40, BL56, BL60	members of AACP; >= 10 yrs of clinical experience	Medication, normal activities
	[Sham] The **same points, non-penetration**	The same	The same
Miyazaki, 2009[43]	One acupoints at the left side of the BL23, Superficially	An acupuncturist with 10 yrs of clinical experience	No
	[Sham]The **same points, non-penetration**	The same	The same
Cherkin, 2009[44]	*Individualized acupuncture*: 74 distinct points were used, half on the "Bladder meridian" that includes points on the back and legs, average 10.8 (5-20) points chosen	6 licensed acupuncturists >=3 yrs of training; 4-19 yrs of experience	A self-care book with information on managing flare-ups, exercise, and life-style modifications.
	Standardized acupuncture: included 8 acupoints commonly used for CLBP (Du 3, Bladder 23- bilateral, low back ashi point, Bladder 40-bilateral, Kidney 3-bilateral) on the low back and leg	The same	The same
	[Sham] a toothpick in a needle guidetube, **points the same, non-penetration**	The same	The same
Haake, 2007[46]	NM	licensed acupuncturists >=140 hours training; 2-36 yrs (median, 8 yrs) experience	NASAID (<=2 days/wk during therapy period, <=1 day/wk during follow-up), other additional therapies were prohibited
	[Sham] avoiding all known verum points or meridians; needles, **superficial**, points **not the same**	The same	The same

Itoh, 2006[47]	This was identified in accessible muscles ideally by the presence of a tender taut band, patient recognition of pain, and local twitch response.	An acupuncturist with 4 yrs of training; 7 yrs of clinical experience.	Medication (as usual)
	[Sham] **Other points**, **Non-penetration**	The same	The same
Inoue, 2006[48]	The point most painful, only LBP in a limited area, which was exacerbated in particular postures	licensed acupuncturists, NM	NM
	[Sham] The **same point**, tube without needle, **non-penetration**	The same	NM
Brinkhaus, 2006[49]	>=4 local points: bladder 20 to 34; bladder 50 to 54; gallbladder 30; governing vessel 3, 4, 5, and 6; and extraordinary points Huatojiaji and Shiqizhuixia, at least 2 distant points: small intestine 3; bladder 40, 60, and 62; kidney 3 and 7; gallbladder 31, 34, and 41; liver 3; and governing vessel 14 and 20. Ear and trigger points	licensed acupuncturists >=140 (median, 350) hours training; >=3 yrs (median, 10 yrs) experience	NSAID, other additional therapies were prohibited
	[sham] At least 6 of 10 predefined **non-acupuncture points**, not in the area of the lower back where the patients were experiencing pain. **Superficial** insertion	The same	The same
Molsberger, 2002[50]	Lumbar region: urinary bladder 23, 25, and gallbladder 30; Lower extremity: were urinary bladder 40, 60 and gallbladder 34; TrPs: four points of maximum pain 'Ahshi points'.	An experienced medical doctor who had studied acupuncture in China (Beijing).	Combination of conventional orthopedic therapy, NSAID
	[sham] at defined **non-acupuncture points** of the lumbar region, and five needles on either side of the back.	The same	The same
Guerra, 2004[34]	Standardized acupoints: 2 local points (LI15, TE14), 2 distal points (GB34, Zhongping Extra point 1–2 cm below Zusanli ST 36), and individualized points. Dense disperse waves of 5-10 Hz at sufficient intensity	Two licensed acupuncturists with >= 4 years of experience	Diclofenac and famotidine pills
	[sham] The **same point, non-penetration, dummy stimulation**	The same	The same
Leibing, 2002[51]	21 fixed body acupoints (nine bilateral, two single points): GV3, 4, BL23, 25, 31, 32, 40, 60, GB34, SP6, Yautungdien； and 6 on the ear (alternately on one ear)	A experienced Taiwanese physician who obtained degrees at the University for Chinese Culture	NSAID as usual
	[sham] Needles were inserted **superficially**, 10–20 mm **distant to** the verum-acupoints, outside the meridians and were not stimulated (no 'de qi'). Superficial, non-acupoints	The same	The same

Mendelson, 1983[52]	for localized low back pain, points 23, 25, 36, and 40 on the urinary bladder meridian; if sciatica was present, points 30,34,39, and 60 on the gallbladder meridian were also used	A surgeon trained at the Chinese Traditional Medical Research Institute in Peking.	NM
	[sham] The **same points**, intradermal injection of 2 percent lidocaine, at non-acupuncture, non-tender sites in lumbar area; needles were inserted **superficially**	The same	NM
Horng, 2013[53]	electrostimulation to the C- and F-points of disease corresponding meridians; set at a frequency of 100 Hz for reduction mode and 40 Hz for enhancement mode. most intense tolerable electrical sensation without muscle contractions	NM	NSAID as usual
	[sham] The **same acupoints**, **no electrostimulation**	NM	The same
Mavrommatis, 2012[54]	the local points ST36, SP9, SP10, GB34, Ex-LE 2, and Ex-LE5; the distal points Li4, Ki3, ST40, and SP6. Starting from the third session, the ES-160 electrostimulator ITO co. (2–6 Hz, 150 milliseconds for 20 minutes) was used to stimulate the needles in pairs ST36-SP9 and GB34-SP10.	A licensed acupuncturist	Etoricoxib tablet (60 mg/d for 60 days, NSAID)
	[sham] The **same points**, retractable needles, **non-penetration**, electrodes simulation.	The same	The same
Lu, 2010[56]	Five acupoints, namely yanglinquan (GB 34), yinglinquan (SP 9), xuehai (SP 10), liangqiu (SP 34) and zhusanli (ST 36); electrostimulation (0.5 mA, a frequency of 2 Hz, 1 ms square pulse at a maximal tolerable intensity)	An experienced acupuncturist	NM
	[sham] 1 cm **left to** the same points; **penetration**, no electrostimulation	The same	NM
Suarez, 2010[55]	Xi Yan, He Ding, GB 34, SP 6, SP 9, Ear-Knee, 1-2 tender Ashi points proximal to the knee; emit a dense disperse (DD) wave impulse at 50 Hz, dispersing at 15 Hz, 20 cycles/minute. Voltage was increased slowly from 5 V to 60 V until maximal tolerance was achieved. Patients rested for 20' with continuing	Six acupuncturists trained in traditional Chinese medicine, licensed by the Texas State Board of Medical Examiners; at least 2 yrs of clinical experience.	Medication
	[sham] Points not relevant to the knee, located in between meridians, 1.5 cm **lateral** to real points, penetration, **superficially**; instead of DD, a 40 Hz adjustable (ADJ) wave, Voltage was increased until the patient could feel it and then immediately turned off.	The same	The same

	Patients rested for 20’ with the needles retained		
Jubb, 2008[58]	LI 4, SP 10, Xiyan (‘Eyes of the knee’), SP9, GB 34, ST 36, LIV 3, BL40, and BL 57. Electrostimulation: two Xiyan points, SP9 and GB34, and BL40 and BL57. Low frequency (2-10 Hz), square-wave pulse of millisecond, Voltage at max tolerate level, 10 min	NM	NSAID, usual analgesia
	[sham] The **same points**, needle retracts into the handle with **non-penetration**. No electrical current.	NM	The same
Itoh, 2008[59]	Lower region standard acupoints: ST34, 35, 36, SP9, 10, GB34	Two acupuncturists with 4 yrs of training; 3 and 8 yrs of experience	NM
	Local trigger points	The same	NM
	[sham] The same trigger points, non-penetration	The same	NM
Foster, 2007[60]	NM	NM	Advice and exercise
	[sham] The **same points**, needle retracts into the handle with **non-penetration**.	NM	The same
Witt, 2005[62]	Semi-standardized, >=6 local points: stomach 34, 35, 36; spleen 9, 10; bladder 40; kidney 10; gall bladder 33, 34; liver 8; extraordinary points Heding, Xiyan. Physicians selected >=2 distant points: spleen 4, 5, 6; stomach 6; bladder 20, 57, 58, 60, 62; kidney 3.	Trained >=140 hours, experienced in acupuncture, have an investigator meeting	NSAID
	[sham] **Non-acupuncture points** (not in the knee area, at least 8 out of 10 points)**, superficially**	The same	The same
Vas, 2004[63]	Local points: GB34, SP9, EX-LE5, and ST36; Distal points: KI3, SP6, LI4, ST40. electrostimulation	An acupuncturist accredited by the Beijing University of Medical Sciences (China); NM	NSAID (diclofenac), used strictly for all patients included
	[sham] The **same points, non-penetration, electrostimulation**	The same	The same
Berman, 2004[64]	5 local points: Yanglinquan [GB34], Yinlinquan [SP9], Zhusanli [ST36], Dubi [ST35], and extra point Xiyan), 4 distal points: Kunlun [UB60], Xuanzhong [GB39], Sanyinjiao [SP 6], and Taixi [Ki3] on meridians that traverse the area of pain; electrostimulation at Xiyan, 8Hz, 0.5 ms pulse width, 20 min	7 acupuncturists were state-licensed; had >= 2 years of clinical experience.	NSAID

	[sham] The **same points, non-penetration, sham** electrostimulation	The same	The same
Takeda, 1994[65]	ST-35, GB-34, SP-9, Extra–31, 32		
	[sham] **1 inch from** the IG points, **superficial insertion**		
Gaw, 1975[67]	At sites of the body corresponding to traditional Chinese acupuncture points	A licensed physician, received acupuncture training in Hong Kong and Taiwan	No (stop usual medications)
	[sham] **Lateral** to same points, **penetration**	The same	The same
Fink, 2001[66]	GB-30, 31, 34, BL-37, ST-40, BL-54, six 'ah shi' points	a physician with sound knowledge of traditional acupuncture techniques	NM
	[sham] At least 5 cm **away from** IG points, **insertion** at **same depth**	The same	NM
Scharf, 2006[61]	Standardized acupoints: Unilateral: ST34, ST36, Xiyan, SP9, SP10, GB34; Unilateral: 1–4 Ahshi points; bilateral according: 1–2 of 16 defined distant points	The 320 physicians were selected from a group of experienced primary care practitioners participating in a large cohort study on acupuncture	Diclofenac, 150 mg/d; Physiotherapy 6 sessions
	[sham] Bilateral, no proximity to traditional acupoints: 3 at the lower limb, 1 at the upper limb, and 1 at the arm, **different acupoint, superficially**	The same	The same
Ashin, 2009[57]	6 acupoints: 5 local points: ST34, ST35, ST36, Liv8, SP10; 1 distal point: ST44; with maximum tolerable intensity of current and a frequency of 3 Hz.	A qualified acupuncturist	Acetaminophen (2 tablets)
	[sham] **Different** types of controls (sham or minimal acupuncture), random points at least 5 cm away from standard points and 3 cm away from meridians and not in the same dermatome, with a depth of no more than 5-10 cm. (**superficially**)	The same	The same
Zanette, 2008[68]	EX 1, PC6, IG4, EX 28, CV 12, CV 6, ST 36, SP 6, and LV 3; UB 20, UB 22, UB 23, GV 4, GV 14, UB 11, and UB60.	NM, NM	Medication (NSAID, anesthesia) as usual

	[sham] **Non-acupuncture points, superficially**	NM, NM	The same
Tam, 2007[69]	6 acupoints: Quchi (LI11), Waiguan (TE5), Hegu (LI4), Zusanli (ST36), Yanglingquan (GB34), Xuanzhong (GB39); Electrostimulation (dense 4 Hz and disperse 20 Hz, started 10 min after insertion until withdrawal), and manipulation	A registered acupuncturist; practicing acupuncture for >= 10 years.	Medication (Methotrexate, NSAID, analgesia) as usual
	The same points, just manipulation	The same	The same
	[sham] The **same points**, **superficially**, **not any stimulation**	The same	The same
Molsberger, 2010[31]	1-3 locus Ahshi points; local and distal points: Lung 1,2, LI4, 11, 14, 15, Sanjiao 5, 13, 14, SI 3,9; additional distal points: ST38, GB34, BL58	31 orthopedists who all had passed nationally recognized acupuncture examinations with a minimum of 140 training hours	NSAID
	[sham] Eight **Non-acupuncture points, superficially** (<=5 mm)	The same	The same
Goldman, 2008[38]	2 Standardized points: LI3, LI4; 20 individualized points: NM; 5-8 additional points: LI5, P5, 6, 7, TW5; Some Trigger points.	8 licensed acupuncturists; average of experience 10 years (range 2 to 26 years)	Medication (NSAID), other physical therapy in progress
	[sham] **The same points,** retractable needles, **non-penetration**	The same	The same
Fink, 2002[39]	Local points: LI4, SJ5; Trigger points: LI10, 11, Lu5	One physician with sound knowledge of traditional acupuncture techniques	No
	[sham] **5 cm lateral** to TG points and meridians and trigger points, **penetration** the same depth	The same	No
Molsberger, 1994[40]	At a non-segmental distal points on the homolateral leg: GB34 (yanglingquan)	A orthopedic doctor trained in Chinese acupuncture	No current therapy with pain killers
	[sham] A point **1.5 cm lateral to (different** from TG)third thoracic vertebra acupoint: UB13 (Feishu); **non-penetration, slight manipulation**	The same	The same
Harris, 2009[70]	Unilateral left: LI11, ST36, SP6, GB34; Right: LI4, LR3, GV20; Ear point: shenmen	NM, NM	Medication

	[sham] **The same points, non-penetration**	The same	The same
Harris, 2008[71]	The same as Harris 2009	1 acupuncturist was trained; 6 years of experience	None
	[sham] **The same points, non-penetration**	The same	The same
Harris, 2005[73]	The same as Harris 2009	2 licensed acupuncturists; 17 years of experience in acupuncture; 95% tx performed by 1 acupuncturist	Normal treatment as usual
	[sham] **same** points, **penetration** the **same depth** as TG, no manipulation	The same	The same
	[sham] **different (irrelevant)** points, **penetration** the same depth, not on relevant meridians, manipulation		
	[sham] **different (irrelevant)** points, **penetration** the same depth, not on relevant meridians, no manipulation		
Martin, 2006[72]	Standardized, Bilaterally, LI4, ST36, LR2, SP6, PC6, HT7; plus 3 cervical and 4 lumber axial on BL channel but did not state actual points, Electrostimulation(2 Hz, 12 Hz at LI4 and ST36)	2 acupuncturists, NM	1.5 days of education
	[sham] The **same points**, **non-penetration, sham electrostimulation**	The same	The same
Assefi, 2005[74]	Alternating between LI11, SP9, CV12, ST25, KI7, TE5, Ex-HN-3 (Yin Tang), KI7, BL17, 18, 20, 22, 43, 44	8 acupuncturists were trained; 15 years of experience	Normal treatment as usual, medication (NSAID)
	[sham] 3 sham acupuncture treatments: acupuncture for an unrelated condition; needle insertion at non-acupoint locations; or non-insertive simulated acupuncture	The same	The same
Tekin, 2013[75]	Bilaterally, trigger points on the back	One acupuncturist, NM, NM	Paracetamol only
	[sham] The **same trigger points**, **non-penetration**	The same	The same
Couto, 2013[76]	The number of muscles with trigger points (mean 4.33); 77% of the points on the upper half of the body, no electrostimulation	A trained acupuncturist with 18 yrs of experience	Analgesics

	[sham] **Trigger points**, sham electroacupuncture, **no current**, **non-penetration**	The same	The same
Chou, 2011[77]	Two acupoints on the according hand: TE-5 (Waiguan), LI11 (Quchi); ipsilateral remote acupuncture points	A well-trained licensed acupuncturist	No
	[sham] The **same points, non-penetration**	The same	No
Tsai, 2010[78]	Selected active TrP in the painful upper trapezius muscle and the latent TrP of the extensor carpi radialis longus muscle	A physiatrist who had experience in TrP injection for 10 yrs	NM
	[sham] The **same points, penetration superficially**	The same	NM
Sun, 2010[79]	Bilaterally, Fenhchi (GB20), Jianliao (TE14), Houxi (SI3)	One Chinese medicine doctor, with >= 5 years of acupuncture experience	NM
	[sham] The **same points, penetration superficially**	The same	NM
Shen, 2009[80]	Only one point used: Hegu LI4 at the left hand	One state-certified dental acupuncturist.	No
	[sham] **1 cm laterally** to same point**, non-penetration**	The same	No
Chou, 2009[81]	Two acupoints on the according hand: TE-5 (Waiguan), LI11 (Quchi); ipsilateral remote acupuncture points	A well-trained licensed acupuncturist	No
	[sham] The **same points, non-penetration**	The same	No
Shen, 2007[82]	Only one point used: Hegu LI4 at the left hand	One state-certified dental acupuncturist.	No
	[sham] **1 cm laterally** to same point**, non-penetration**	The same	No
Goddard, 2002[83]	4 points, bilaterally, Hegu (LI4), ST6	A certified dental acupuncturist	No
	[sham] **1 cm laterally** to same point**, penetration superficially**	The same	No
Birch, 1998[84]	Relevant acupoints, Shallowly (2-3 mm) inserted in two sequential stages, first stage (10 min): bilaterally hands and feet, SI3, BL62, GB41, TE5; second stage (10 min): bilaterally neck, shoulder and upper back, GB12, 20, 21, BL10, 11, Unilaterally GV14	A licensed acupuncturist; 13 years of experience	Infrared lamp

	[sham] **Irrelevant acupoints**, **Shallowly (2-3 mm) inserted** in two sequential stages, first stage (10 min): bilaterally hands and feet, LI5, GB42, TW8, ST41; second stage (10 min): bilaterally neck, shoulder and upper back, BL16, SI9, LI15	The same	Sham infrared lamp
McMilland, 1997[85]	Selected active TrP in the painful jaw muscle	NM	A drop of isotonic saline was injected TrP selected.
	[sham] The **same points, superficially**	NM	The same
Diracoglu, 2012[87]	Selected active TrP in the painful muscles, intramuscular stimulation	NM	NM
	[sham] Areas **away from** selected active TrP, **intracutaneous** stimulation	NM	NM
Smith, 2007[86]	Only one acupoints was used: ST7, bilaterally	A experienced clinician	Treatment as usual
	[sham] The **same point, non-penetration**	The same	The same

CT: controlled trials; D: Distal; L: Local; IG, intervention group; M: Manual; Mth: month; NM: not mentioned; TI: Textbooks information; TrP: Trigger point; TCM: traditional Chinese Medicine; yr: year.
Meridians of acupuncture: EX, extra point; CV, conception vessel; LI, large intestine; L, Lung; GV, governor vessel; LV, liver; PC, pericardium; SP, spleen; ST, stomach; B, bladder; BL, bladder; TE, Triple Energizer; H, Heart.

Table S8. Reasons for trials excluded

Study		Reason
Irnich, 2002[1]	NP	This was a crossover design trial; the data at first stage were not showed.
Carlsson, 2001[2]	LBP	Relevant follow-up data or figures were not showed.
Macdonald, 1983[3]	LBP	Relevant follow-up data or figures were not showed.
Gunn, 1980[4]	LBP	Relevant follow-up data or figures were not showed.
Foster, 2010[5]	OA (keen)	Relevant follow-up data or figures were not showed.
Fink, 2002[6]	Chronic epicondylitis	Secondary analysis of original article, published at the same year
David, 1999[7]	RA	This was a crossover design trial; the data at first stage were not showed.
Yao, 2012[8]	Carpal Tunnel Syndrome	Relevant follow-up data were not showed.
Hubshcer, 2008[9] Germany	Muscle soreness	Relevant follow-up data or figures were not showed.
Barlas, 2000[10]	Muscle soreness	Relevant follow-up data or figures were not showed.

LBP, low back pain; NP, neck pain; OA, osteoarthritis; RA, rheumatoid arthritis

1 Irnich, D. *et al.* Immediate effects of dry needling and acupuncture at distant points in chronic neck pain: results of a randomized, double-blind, sham-controlled crossover trial. *Pain* **99**, 83-89 (2002).

2 Carlsson, C. P. & Sjolund, B. H. Acupuncture for chronic low back pain: a randomized placebo-controlled study with long-term follow-up. *The Clinical journal of pain* **17**, 296-305 (2001).

3 Macdonald, A. J., Macrae, K. D., Master, B. R. & Rubin, A. P. Superficial acupuncture in the relief of chronic low back pain. *Annals of the Royal College of Surgeons of England* **65**, 44-46 (1983).

4 Gunn, C. C., Milbrandt, W. E., Little, A. S. & Mason, K. E. Dry needling of muscle motor points for chronic low-back pain. A randomized clinical trials with long-term follow-up. *Spine* **5**, 279-291 (1980).

5 Foster, N. E., Thomas, E., Hill, J. C. & Hay, E. M. The relationship between patient and practitioner expectations and preferences and clinical outcomes in a trial of exercise and acupuncture for knee osteoarthritis. *European journal of pain (London, England)* **14**, 402-409, doi:10.1016/j.ejpain.2009.06.010 (2010).

6 Fink, M. *et al.* Chronic epicondylitis: effects of real and sham acupuncture treatment: a randomised controlled patient- and examiner-blinded long-term trial. *Forschende Komplementarmedizin und klassische Naturheilkunde = Research in complementary and natural classical medicine* **9**, 210-215, doi:66030 (2002).

7 David, J., Townsend, S., Sathanathan, R., Kriss, S. & Dore, C. J. The effect of acupuncture on patients with rheumatoid arthritis: a randomized, placebo-controlled cross-over study. *Rheumatology (Oxford, England)* **38**, 864-869 (1999).

8 Yao, E. *et al.* Randomized controlled trial comparing acupuncture with placebo acupuncture for the treatment of carpal tunnel syndrome. *PM & R : the journal of injury, function, and rehabilitation* **4**, 367-373, doi:10.1016/j.pmrj.2012.01.008 (2012).

9 Hubscher, M., Vogt, L., Bernhorster, M., Rosenhagen, A. & Banzer, W. Effects of acupuncture on symptoms and muscle function in delayed-onset muscle soreness. *Journal of alternative and complementary medicine (New York, N.Y.)* **14**, 1011-1016, doi:10.1089/acm.2008.0173 (2008).

10 Barlas, P., Robinson, J., Allen, J. & Baxter, G. D. Lack of effect of acupuncture upon signs and symptoms of delayed onset muscle soreness. *Clinical physiology (Oxford, England)* **20**, 449-456 (2000).

Table S9. Data converted and data extracted from figures

Study	Condition	Reason
Miyazaki, 2009 [43]	LBP	Data were acquired from figures, and the difference between groups was the same as the results in the original article.
Ashin, 2009 [57]	OA (keen)	Data were acquired from figures, and the difference between groups was the same as the results in the original article.
Itoh, 2008 [59]	OA (keen)	Data were acquired from figures, and the difference between groups was the same as the results in the original article.
Fink, 2001 [66]	OA (hip)	The data about pain were not acquired directly from original article, but from a systematic review.
Gaw, 1975 [67]	OA (keen, hip, lumbar, thoracic, neck, finger)	The data about pain were ranked data, so we converted them into continuous variable; and the difference between groups was the same as the results in the original article.
Sun, 2010 [79]	Myofascial pain	Mean and its SD was calculated from median and its interquartile range
Smith, 2007 [86]	Myofascial pain	The standard deviation was calculated from P value.

LBP, low back pain; OA, osteoarthritis; SD, standard deviation

Table S10. Risk of Bias.

Author and year	Q1. Randomization Adequate?	Q2. Treatment Allocation Concealed?	Q3. Groups similar at baseline re: prognostic indicators	Q4. Patient blinded to the intervention?	Q5. Care provider blinded to the intervention?	Q6. Outcome assessor blinded to the intervention?	Q7. Co-interventions avoided or similar?	Q8. Compliance acceptable in all groups	Q9. Dropout rate describe d and acceptable?	Q10. Timing of the outcome assessment in all groups similar?	Q11. Analysis includes an intention-to-treat analysis?	Q12. Reports of the study free of suggestion of selective outcome reporting?	Total score
Neck Pain													
Liang, 2011[25]	Y	Y	Y	Y	N	N	Y	?	Y	Y	N	Y	8
Sahin, 2010[26]	Y	Y	Y	Y	N	Y	?	?	Y	Y	N	N	7
Itoh, 2007[27]	Y	?	Y	Y	N	Y	Y	?	N	Y	N	Y	7
Zhu, 2002[28]	?	?	Y	Y	N	N	?	?	Y	Y	Y	Y	5
Cervical Spondylosis													
Fu, 2009[29]	Y	Y	Y	Y	N	?	Y	Y	Y	Y	N	Y	9
Whiplash													
Tough, 2010[30]	Y	Y	Y	Y	N	Y	?	?	N	Y	N	Y	9
Shoulder pain													
Molsberger, 2010[31]	Y	Y	Y	Y	N	Y	?	N	N	Y	Y	Y	7
Lathia, 2009[32]	Y	Y	?	Y	N	Y	N	?	Y	Y	N	N	6

Dyson, 2007[33]	?	?	Y	Y	N	Y	Y	?	Y	Y	N	N	6
Guerra, 2004[34]	Y	Y	Y	Y	N	Y	Y	?	Y	Y	N	Y	8
Kleinhenz, 1999[35]	Y	Y	N	Y	N	Y	Y	?	N	Y	N	N	6
Neck and Shoulder Pain													
He, 2004[36]	Y	?	Y	Y	N	Y	?	Y	Y	Y	Y	N	8
Nabeta, 2002[37]	Y	?	Y	Y	N	?	?	Y	Y	Y	Y	N	7
Arm pain													
Goldman, 2008[38]	Y	Y	Y	Y	N	Y	?	Y	Y	Y	N	Y	9
Fink, 2002[39]	Y	?	Y	Y	N	Y	Y	?	Y	Y	N	Y	8
Molsberger, 1994[40]	?	?	N	Y	N	N	Y	Y	Y	Y	Y	N	6
Back Pain													
Hasegawa, 2013[41]	Y	Y	Y	Y	N	Y	Y	Y	Y	Y	Y	Y	11
Vas, 2012[42]	Y	Y	Y	Y	N	Y	Y	?	N	Y	Y	Y	8
Kennedy, 2008[45]	Y	Y	Y	Y	N	Y	Y	Y	Y	Y	Y	Y	11
Miyazaki, 2009[43]	?	?	Y	Y	N	Y	Y	Y	Y	Y	Y	Y	10
Cherkin, 2009[44]	Y	Y	Y	Y	N	Y	Y	Y	Y	Y	N	N	9
Haake, 2007[46]	Y	Y	Y	Y	N	Y	Y	Y	Y	Y	N	Y	10
Itoh, 2006[47]	Y	Y	Y	Y	N	Y	Y	Y	Y	Y	N	?	11
Inoue, 2006[48]	Y	Y	Y	Y	N	Y	?	Y	Y	Y	Y	N	9

Brinkhaus, 2006[49]	Y	Y	Y	Y	N	Y	Y	?	Y	Y	N	Y	9
Molsberger, 2002[50]	Y	?	Y	Y	N	Y	Y	?	N	Y	N	N	6
Leibing, 2002[51]	Y	?	Y	Y	N	Y	Y	?	N	Y	Y	Y	8
Mendelson, 1983[52]	Y	?	Y	Y	N	Y	N	Y	N	Y	N	N	6
Knee osteoarthritis													
Horng, 2013[53]	Y	Y	Y	N	N	Y	Y	Y	N	Y	N	Y	8
Mavrommatis, 2012[54]	Y	?	Y	Y	N	Y	Y	Y	Y	Y	N	Y	9
Lu, 2010[56]	?	?	Y	Y	N	N	?	Y	Y	Y	Y	N	6
Suarez, 2010[55]	Y	Y	Y	Y	N	Y	?	?	Y	Y	Y	Y	9
Jubb, 2008[58]	Y	?	Y	Y	N	Y	Y	Y	Y	Y	N	N	8
Itoh, 2008[59]	?	?	Y	Y	N	Y	?	?	N	Y	N	Y	5
Foster, 2007[60]	Y	?	?	Y	N	Y	Y	?	Y	Y	N	Y	7
Scharf, 2006[61]	Y	Y	Y	Y	N	?	Y	?	N	Y	N	N	6
Witt, 2005[62]	Y	Y	Y	Y	N	Y	Y	?	Y	Y	N	Y	9
Vas, 2004[63]	Y	Y	Y	Y	N	Y	Y	Y	Y	Y	N	Y	10
Berman, 2004[64]	Y	Y	Y	Y	N	Y	Y	N	N	Y	N	Y	8
Takeda, 1994[65]	Y	Y	Y	Y	N	Y	Y	?	Y	Y	?	Y	9
Ashin, 2009[57]	?	?	Y	Y	N	N	Y	?	Y	Y	N	Y	6

Hip osteoarthritis													
Fink, 2001[66]	Y	?	Y	Y	N	Y	?	?	Y	Y	N	Y	7
Gaw, 1975[67]	?	?	?	Y	N	Y	Y	?	Y	Y	Y	N	6
Rheumatoid osteoarthritis													
Zanette, 2008[68]	Y	?	Y	Y	N	Y	Y	?	N	Y	Y	Y	8
Tam, 2007[69]	Y	Y	Y	Y	N	Y	Y	?	N	Y	Y	Y	9
Fibromyalgia													
Harris, 2009[70]	Y	Y	?	Y	N	Y	Y	Y	Y	Y	Y	N	9
Harris, 2008[71]	Y	Y	?	Y	N	Y	Y	Y	Y	Y	N	N	8
Harris, 2005[73]	Y	Y	Y	Y	N	Y	?	?	N	Y	N	Y	8
Martin, 2006[72]	?	Y	Y	Y	N	Y	Y	Y	Y	Y	N	Y	10
Assefi, 2005[74]	Y	Y	Y	Y	N	Y	?	Y	Y	Y	N	Y	9
Myofascial Pain													
Tekin, 2013[75]	Y	?	Y	Y	N	Y	Y	?	N	Y	N	N	6
Couto, 2013[76]	?	Y	Y	Y	N	Y	Y	?	Y	Y	Y	Y	9
Chou, 2011[77]	?	?	Y	Y	N	Y	Y	Y	Y	Y	Y	N	8
Tsai, 2010[78]	Y	?	Y	Y	N	Y	Y	Y	Y	Y	Y	N	9

Sun, 2010[79]	Y	?	Y	?	N	Y	?	?	Y	Y	Y	N	6
Shen, 2009[80]	Y	?	Y	Y	N	Y	Y	Y	Y	Y	N	N	8
Chou, 2009[81]	Y	?	Y	Y	N	Y	Y	Y	Y	Y	Y	N	9
Shen, 2007[82]	?	?	Y	Y	N	Y	Y	Y	Y	Y	Y	N	8
Goddard, 2002[83]	Y	?	Y	Y	N	Y	Y	Y	Y	Y	Y	N	9
Birch, 1998[84]	?	?	Y	Y	N	Y	N	?	N	Y	?	N	4
McMilland, 1997[85]	?	?	?	Y	N	Y	Y	Y	Y	Y	Y	N	7
Diracoglu, 2012[87]	Y	?	Y	Y	N	Y	?	?	Y	Y	N	N	6
Smith, 2007[86]	Y	Y	N	Y	N	Y	?	Y	Y	Y	Y	N	8

ITT indicates intention to treat; NA, not applicable (in a none-time intervention, such as surgery, compliance is not an issue); RCTs, randomized clinical trials; Y, yes; N, no or unsure. The Cochrane risk of bias tool: the number of participants who were included in the study but did not complete the observation period or were not included in the analysis must be described and reasons given. If the percentage of withdrawals and drop-outs does not exceed 15% for short-term follow-up and does not lead to substantial bias a “yes” is scored. (N.B. these percentages are arbitrary, not supported by the literature).

Table S11 - Checklist of items to include when reporting a systematic review or meta-analysis

Section/topic	#	Checklist item	Reported on page #
**TITLE**			
Title	1	Identify the report as a systematic review, meta-analysis, or both.	1
**ABSTRACT**			
Structured summary	2	Provide a structured summary including, as applicable: background; objectives; data sources; study eligibility criteria, participants, and interventions; study appraisal and synthesis methods; results; limitations; conclusions and implications of key findings; systematic review registration number.	2
**INTRODUCTION**			
Rationale	3	Describe the rationale for the review in the context of what is already known.	2-3
Objectives	4	Provide an explicit statement of questions being addressed with reference to participants, interventions, comparisons, outcomes, and study design (PICOS).	4
**METHODS**			
Protocol and registration	5	Indicate if a review protocol exists, if and where it can be accessed (e.g., Web address), and, if available, provide registration information including registration number.	4
Eligibility criteria	6	Specify study characteristics (e.g., PICOS, length of follow-up) and report characteristics (e.g., years considered, language, publication status) used as criteria for eligibility, giving rationale.	4-6
Information sources	7	Describe all information sources (e.g., databases with dates of coverage, contact with study authors to identify additional studies) in the search and date last searched.	6
Search	8	Present full electronic search strategy for at least one database, including any limits used, such that it could be repeated.	6
Study selection	9	State the process for selecting studies (i.e., screening, eligibility, included in systematic review, and, if applicable, included in the meta-analysis).	6-7

Section/topic	#	Checklist item	Reported on page #
Data collection process	10	Describe method of data extraction from reports (e.g., piloted forms, independently, in duplicate) and any processes for obtaining and confirming data from investigators.	7
Data items	11	List and define all variables for which data were sought (e.g., PICOS, funding sources) and any assumptions and simplifications made.	7
Risk of bias in individual studies	12	Describe methods used for assessing risk of bias of individual studies (including specification of whether this was done at the study or outcome level), and how this information is to be used in any data synthesis.	8
Summary measures	13	State the principal summary measures (e.g., risk ratio, difference in means).	7
Synthesis of results	14	Describe the methods of handling data and combining results of studies, if done, including measures of consistency (e.g., I^2) for each meta-analysis.	8-10
Risk of bias across studies	15	Specify any assessment of risk of bias that may affect the cumulative evidence (e.g., publication bias, selective reporting within studies).	10-11
Additional analyses	16	Describe methods of additional analyses (e.g., sensitivity or subgroup analyses, meta-regression), if done, indicating which were pre-specified.	8-10
RESULTS			
Study selection	17	Give numbers of studies screened, assessed for eligibility, and included in the review, with reasons for exclusions at each stage, ideally with a flow diagram.	11
Study characteristics	18	For each study, present characteristics for which data were extracted (e.g., study size, PICOS, follow-up period) and provide the citations.	12-13
Risk of bias within studies	19	Present data on risk of bias of each study and, if available, any outcome-level assessment (see Item 12).	13
Results of individual studies	20	For all outcomes considered (benefits or harms), present, for each study: (a) simple summary data for each intervention group and (b) effect estimates and confidence intervals, ideally with a forest plot.	13-22

Section/topic	#	Checklist item	Reported on page #
Synthesis of results	21	Present results of each meta-analysis done, including confidence intervals and measures of consistency.	13-22
Risk of bias across studies	22	Present results of any assessment of risk of bias across studies (see Item 15).	13
Additional analysis	23	Give results of additional analyses, if done (e.g., sensitivity or subgroup analyses, meta-regression [see Item 16]).	13-22
DISCUSSION			
Summary of evidence	24	Summarize the main findings including the strength of evidence for each main outcome; consider their relevance to key groups (e.g., health care providers, users, and policy makers).	22
Limitations	25	Discuss limitations at study and outcome level (e.g., risk of bias), and at review level (e.g., incomplete retrieval of identified research, reporting bias).	28-30
Conclusions	26	Provide a general interpretation of the results in the context of other evidence, and implications for future research.	31
FUNDING			
Funding	27	Describe sources of funding for the systematic review and other support (e.g., supply of data); role of funders for the systematic review.	32

Figure S1 Acupuncture for musculoskeletal pain for all conditions.

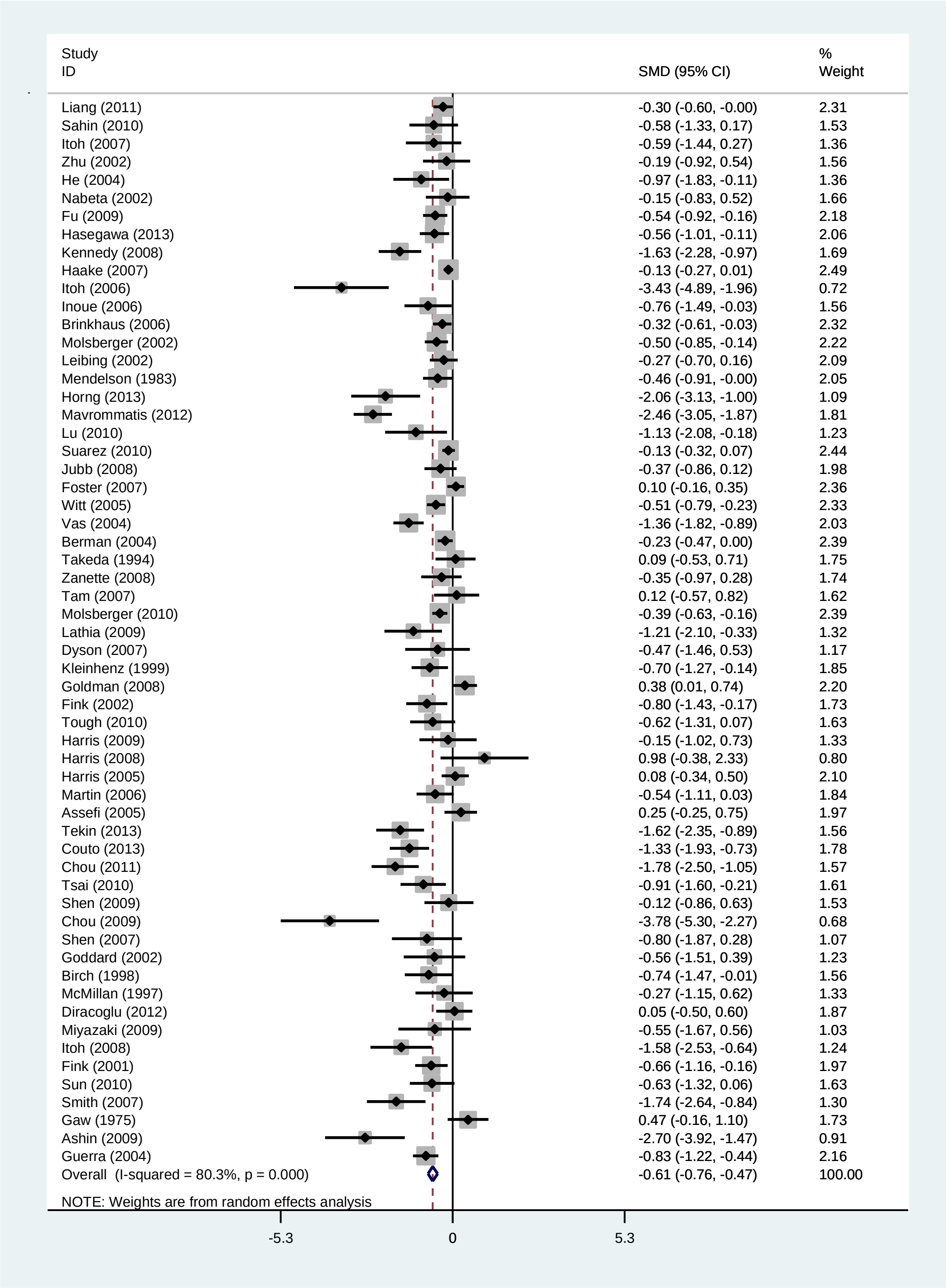

Figure S2 Acupuncture for musculoskeletal disability for all conditions.

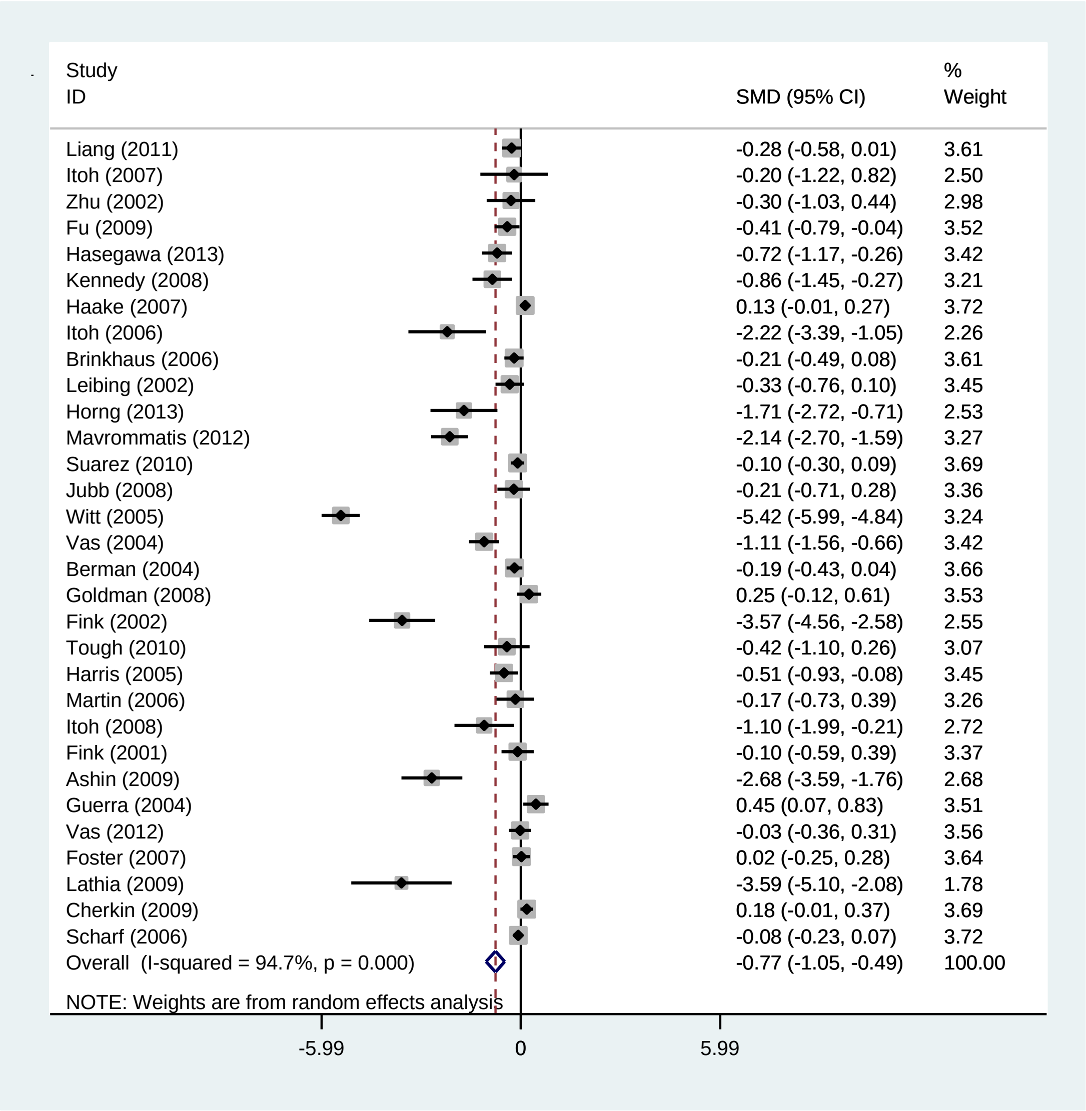

Supplement: Supplementary Information [file srep30675-s1.pdf]
